# Genetic turnovers and northern survival during the last glacial maximum in European brown bears

**DOI:** 10.1002/ece3.5172

**Published:** 2019-04-16

**Authors:** Erik Ersmark, Gennady Baryshnikov, Thomas Higham, Alain Argant, Pedro Castaños, Doris Döppes, Mihaly Gasparik, Mietje Germonpré, Kerstin Lidén, Grzegorz Lipecki, Adrian Marciszak, Rebecca Miller, Marta Moreno‐García, Martina Pacher, Marius Robu, Ricardo Rodriguez‐Varela, Manuel Rojo Guerra, Martin Sabol, Nikolai Spassov, Jan Storå, Christina Valdiosera, Aritza Villaluenga, John R. Stewart, Love Dalén

**Affiliations:** ^1^ Department of Bioinformatics and Genetics Swedish Museum of Natural History Stockholm Sweden; ^2^ Department of Zoology Stockholm University Stockholm Sweden; ^3^ Zoological Institute Russian Academy of Sciences St Petersburg Russia; ^4^ Research Laboratory for Archaeology and the History of Art University of Oxford Oxford UK; ^5^ CNRS, Minist. Culture, LAMPEA, UMR 7269 Aix Marseille University Aix‐en‐Provence France; ^6^ Geo‐Q Aranzadi Society of Sciences Leioa Spain; ^7^ Reiss‐Engelhorn Museen Mannheim Germany; ^8^ Department of Palaeontology and Geology Hungarian Natural History Museum Budapest Hungary; ^9^ Operational Direction “Earth and History of Life” Royal Belgian Institute of Natural Sciences Brussel Belgium; ^10^ Department of Archaeology and Classical Studies Stockholm University Stockholm Sweden; ^11^ Institute of Systematics and Evolution of Animals Polish Academy of Sciences Kraków Poland; ^12^ Department of Paleozoology, Institute of Evolutionary Biology and Ecology, Faculty of Biological Sciences University of Wrocław Wrocław Poland; ^13^ Service of Prehistory University of Liège Liège Belgium; ^14^ GI Arqueobiología, Instituto de Historia Consejo Superior de Investigaciones Científicas Madrid Spain; ^15^ Institute of Palaeontology University of Vienna Vienna Austria; ^16^ “Emil Racoviţă” Institute of Speleology Romanian Academy Bucharest Romania; ^17^ Department of Prehistory and Archaeology University of Valladolid Valladolid Spain; ^18^ Department of Geology and Palaeontology, Faculty of Natural Sciences Comenius University Bratislava Slovak Republic; ^19^ National Museum of Natural History at the Bulgarian Academy of Sciences Sofia Bulgaria; ^20^ Department of Archaeology and History La Trobe University Melbourne Victoria Australia; ^21^ Aranzadi Society of Sciences Donostia‐San Sebastian Spain; ^22^ Facultad de Letras, High Yield Research Group on Prehistory University of the Basque Country (UPV‐EHU) Vitoria‐Gasteiz Spain; ^23^ Faculty of Science and Technology Bournemouth University Dorset UK

**Keywords:** LGM, mtDNA, phylogeography, refugia, *Ursus arctos*

## Abstract

The current phylogeographic pattern of European brown bears (*Ursus arctos*) has commonly been explained by postglacial recolonization out of geographically distinct refugia in southern Europe, a pattern well in accordance with the expansion/contraction model. Studies of ancient DNA from brown bear remains have questioned this pattern, but have failed to explain the glacial distribution of mitochondrial brown bear clades and their subsequent expansion across the European continent. We here present 136 new mitochondrial sequences generated from 346 remains from Europe, ranging in age between the Late Pleistocene and historical times. The genetic data show a high Late Pleistocene diversity across the continent and challenge the strict confinement of bears to traditional southern refugia during the last glacial maximum (LGM). The mitochondrial data further suggest a genetic turnover just before this time, as well as a steep demographic decline starting in the mid‐Holocene. Levels of stable nitrogen isotopes from the remains confirm a previously proposed shift toward increasing herbivory around the LGM in Europe. Overall, these results suggest that in addition to climate, anthropogenic impact and inter‐specific competition may have had more important effects on the brown bear's ecology, demography, and genetic structure than previously thought.

## 1 INTRODUCTION

The European fauna has been repeatedly and profoundly affected by climate change during the Late Quaternary. Alternating glacials and interglacials have caused populations to shift in size and distribution, and even to go extinct (Stewart, Lister, Barnes, & Dalen, 2010). The mechanism of climate‐driven fluctuations has been described as the expansion/contraction (E/C) model (Hewitt, 1999,1996), which predicts that populations will become genetically differentiated during refugial isolation and mix as their ranges expand and overlap. The current phylogeographic patterns in Europe, for most temperate species, are accordingly thought to trace back to an expansion after the last glacial maximum (LGM) some 20,000 years before present (cal. B.P.). As postglacial warming provided new habitats at higher latitudes, warm‐adapted animals were able to disperse and expand out of southern refugia, primarily located in the Mediterranean peninsulas (Hewitt, 1999; Taberlet, Fumagalli, Wust‐Saucy, & Cosson, 1998). One of the key species used to illustrate this postglacial expansion, and the E/C model in general, is the European brown bear (*Ursus arctos*; Hewitt, 1999,2004).

The brown bear is one of the most abundant large carnivores in contemporary Europe (Chapron et al., 2014). Despite this fact, its distribution has contracted during historical times due to habitat loss and human hunting (Servheen, Darling, & Archibald, 1990). From having spanned the entire continent during most of the Holocene, the range is currently limited to the forested regions of northern and eastern Europe. In southern and western Europe (central Italy and northern Spain), a few small and endangered populations still prevail (Ciucci & Boitani, 2008; Palomero, Ballesteros, Herrero, & Nores, 2006; Randi, Gentile, Boscagli, Huber, & Roth, 1994). Concern about the conservation of these and other similarly isolated and endangered populations has been one important factor behind the multitude of phylogeographic studies on the brown bear. The results, both from Europe and elsewhere, have in many cases revealed the existence of spatial patterns, not least in the distribution of mitochondrial diversity (Davison et al., 2011; Hirata, Abramov, Baryshnikov, & Masuda, 2014; Taberlet & Bouvet, 1994; Taberlet, Swenson, Sandegren, & Bjarvall, 1995). This has been explained by a strong philopatry observed in female bears, restricting their dispersal and thereby the spread of mitochondrial lineages (Stoen, Zedrosser, Saebo, & Swenson, 2006). The mitochondrial diversity in Europe is divided into a western (clade 1) and an eastern clade (clade 3), meeting at two contact zones: central Scandinavia and the Carpathian mountains (Davison et al., 2011; Kohn, Knauer, Stoffella, Schröder, & Pääbo, 1995; Randi et al., 1994; Taberlet & Bouvet, 1994). Davison et al. (2011) have proposed a further division of the western clade into several subclades, where two of these are thought to represent bears originating from separate glacial refugia: the Iberian Peninsula (subclade 1a) and the Italian–Balkan refugium (subclade 1b), respectively (Davison et al., 2011). The latter has also been proposed based on morphological features in modern bears (Spassov, 1997). The origin of the eastern clade, clade 3, remains more elusive, especially since it is the most widespread mitochondrial clade in contemporary brown bears, occurring all across northern Eurasia and in North America. The Carpathian Mountains and the Caucasus have both been suggested as potential refugia during the LGM, for bears belonging to clade 3 in Europe (Korsten et al., 2009; Sommer & Benecke, 2005).

Although the E/C model and the hypothesis of isolated refugia are supported by the distinct phylogeographic pattern seen in bears across Europe today, ancient DNA from brown bears dating to the Late Pleistocene and Early Holocene has put the model and the hypothesis into question (Valdiosera et al., 2007,2008). In these studies, the mitochondrial sequences obtained from ancient bears instead indicate contact and gene flow between populations in southernmost Europe during the LGM. Moreover, two bears, dated to before the LGM, have yielded mtDNA that contradict the spatial distribution of the two clades in Europe, suggesting no geographical structure before the LGM (Hofreiter et al., 2004).

Recent studies on ancient brown bear remains from Scandinavia and the British Isles have shed more light on the expansion of brown bears in western Europe after the LGM (Bray et al., 2013; Edwards et al., 2011,2014). The presence of clade 3 in northern Scandinavia by the mid‐Holocene suggests that the present latitudinal division, with clades 3 and 1 occupying the north and south, respectively, was present already at this time (Bray et al., 2013). The results from the British Isles show an early expansion of brown bears in England after the LGM, all belonging to subclade 1a (Edwards et al., 2014). Although this does not contradict the southern glacial refugium scenario, such an early appearance in England may be consistent with a hypothesis where bears were present north of the Mediterranean peninsulas during the LGM. In Ireland, a separate lineage (clade 2) was identified in ancient brown bears, apparently obtained through hybridization with polar bears at times when their ranges overlapped here during the Late Pleistocene (Edwards et al., 2011). This introgression of polar bear mitochondria in Europe has so far only been recorded in Ireland, where the youngest bear carrying clade 2 was dated to c. 9,700 cal. B.P. (Edwards et al., 2011). Recently, introgression from cave bears has also been revealed from nuclear data (Barlow et al., 2018).

Ancient DNA studies of brown bears have consequently provided indications of a different phylogeography in the past than that seen today among European brown bears. However, to date, very few brown bear remains dated to the LGM or earlier have been successfully sequenced, especially from the putative refugia in southern Europe. This can to some extent be explained by the lack of available remains suitable for DNA analysis. Although brown bears are fairly common in the Late Pleistocene fossil and archaeological record in southern Europe (Sommer & Benecke, 2005), their preservation is generally poor. At cave sites, where faunal remains are more well‐preserved, Late Pleistocene records are often dominated by remains of the morphologically similar cave bear (*Ursus spelaeus*) (Münzel et al., 2011; Sabol, 2001), which went extinct around 25–30,000 cal. B.P. (Bocherens et al., 2014; Pacher & Stuart, 2009; Stiller et al., 2010). This fact has complicated the identification and retrieval of brown bear material from many of these locations.

Changes in climate and habitat availability might have influenced more than just the genetic distribution of the brown bears. Due to its varied diet, the brown bear is well suited as a model species to study changes in physiological ecology through time. One way of looking at this is by the application of stable isotope analysis. This has previously been done on both modern and ancient bears, mainly to characterize dietary variation in geographically and temporally diverse bear populations (Bocherens et al., 2004; Döppes, Rosendahl, Pacher, & Bocherens, 2010; Hilderbrand et al., 1992; Münzel et al., 2011; Robu et al., 2013).

In this study, we sequenced mitochondrial DNA (mtDNA) from ancient brown bear remains collected from across Europe, in order to study the long‐term phylogeographic changes that took place across the continent during the postglacial expansion, and to trace the mitochondrial lineages across the LGM. We particularly wanted to test the hypothesis that the mitochondrial diversity seen in modern bears is the result of previous isolation in southern refugia. We further wanted to estimate changes in diet over the same period, through the study of stable isotopes, and relate these to ecological changes affecting the species.

## 2 MATERIALS AND METHODS

A total of 346 samples from bear remains were obtained from a wide geographic range within Europe, recovered from museum collections or provided by collaborators. Some remains had previously been subject to morphological and/or isotope analyses (references below Table S1). Many samples lacked a well‐defined age or a proper stratigraphic context. In spite of this, a selection was made with focus on those assumed to date from the Late Pleistocene (Table S1). Teeth and compact bone were preferred where available, to minimize the risk of external contamination of the DNA.

### 2.1 Ancient DNA extraction and amplification

DNA extraction and PCR setup were performed at the Swedish Museum of Natural History (NRM) in Stockholm, in a laboratory exclusively used for ancient DNA. All handling was carried out with sterilized equipment, and strict measures were taken to avoid contamination from exogenous sources or between samples. Mitochondrial DNA was extracted from bones and teeth using methods optimized for ancient DNA (Ersmark et al., 2015). Amplification was designed to obtain DNA sequences compatible with previous studies on ancient brown bears, which mainly have targeted a short but variable stretch of the control region (CR) (Davison et al., 2011). On this basis, a set of primers were chosen to target two sections of the mentioned region, 111 and 135 bp long. Primer pairs targeted both sections, but mainly using shorter overlapping fragments (Table S3).

PCR amplifications were performed in 25 µl reactions, using 2 µl of DNA extract, 1 mM MgCl_2_ (Qiagen), 0.2 mM dNTPs, 0.2 µM of each primer, 1× PCR buffer (Qiagen), 0.1 mg/ml BSA, and 2 units of HotStar Taq (Qiagen). Amplifications started with a 10 min of denaturation step at 95°C, followed by 55 cycles of denaturation at 95°C for 30 s, annealing for 30 s at 58–62°C, followed by extension at 72°C for 30 s. A final extension step at 72°C for 7 min was included at the end of the procedure. Confirming successful amplifications was done using gel electrophoresis, by applying 5 µl PCR product on a 1.5% agarose gel prepared with florescent GelRed (Biotium Inc.). The gel was run in 0.5× TBE buffer and inspected with UV light. The PCR products were further purified with EXO‐SAP enzymes (1:4).

To resolve the effects of DNA damage and monitor contamination, negative controls were routinely incorporated, and amplifications were repeated at least once to confirm the final consensus sequences. Final sequencing was performed on an ABI 3130xl Genetic Analyzer (Applied Biosystems Inc.).

### 2.2 Radiocarbon dating and stable isotope analysis

A subset of 72 brown bear samples that produced PCR products for the two fragments and contained enough material were resampled for collagen. We performed pretreatment and collagen extraction as well as the final radiocarbon dating at the Oxford Radiocarbon Accelerator Unit (ORAU) in Oxford, UK. Two additional samples were submitted to Beta Analytic (London BioScience Innovation Centre) for radiocarbon dating. To avoid surface contaminants, the samples were meticulously cleaned before homogenization. Internal contamination from sedimentary carbonates and humic acids was removed with an acid–base–acid treatment and rinsing in Milli‐Q deionized water. All samples were pretreated employing ultrafiltration (Brown, Nelson, Vogel, & Southon, 1988; Higham, Jacobi, & Ramsey, 2006), and dating was performed using accelerator mass spectrometry (AMS) (Brock, Higham, Ditchfield, & Ramsey, 2010). Measures of stable carbon (δ^13^C) and nitrogen (δ^15^N) isotopes, which can be used to infer diet (Schoeninger & DeNiro, 1984), were also obtained in association with the dating process. These data were compiled, together with published measurements available on ancient brown bears from the British Isles (Edwards et al., 2014; Jacobi & Higham, 2008), Scandinavia (Fornander, Eriksson, & Lidén, 2008; Salmi, Äikäs, Fjellström, & Spangen, 2015), and central/western Europe (Bocherens, 2015; Döppes & Pacher, 2014; Döppes et al., 2008, 2010; Münzel et al., 2011). In order to compare the diet of Late Pleistocene brown bears with contemporary cave bears, a smaller set of published isotope data from the latter species was also included (Fernández‐Mosquera, Vila‐Taboada, & Grandal‐d'Anglade, 2001; Münzel et al., 2011; Stiller et al., 2010).

### 2.3 Phylogenetic analyses

The successfully amplified and confirmed sequences were aligned in Geneious v. 7.1.3 (Kearse et al., 2012). Published equivalents covering or overlapping the same region were added to the alignment, both from modern and ancient European brown bears (GenBank accession numbers in Table S2). Further analyses of the genetic data were performed by construction and interpretation of phylogenetic networks, using the PopART software (Leigh & Bryant, 2015) and isochronous maps of haplotype distributions. To estimate structuring over time, the European population was divided into two geographical bins, among which pairwise differentiation over time was inferred from an AMOVA analysis using Φ_ST_ statistics calculated in GENALEX (Peakall & Smouse, 2012).

To establish intraspecific relationships and estimate expansions and declines in population size, a set of Bayesian analyses were performed in BEAST 1.8.0 (Drummond & Rambaut, 2007). The HKY + Γ model of nucleotide substitution was applied after selection in ModelTest (Posada & Crandall, 1998). A flexible coalescent prior the Bayesian Skygrid (Gill et al., 2013) was used, with a molecular clock estimated from the included radiocarbon dates. Two runs of Markov chain Monte Carlo (MCMC) were performed for 100 million generations each, sampling every 10,000 MCMC generations and excluding the initial 10% as burn‐in. The resulting log and tree files were combined using LogCombiner v.1.8.0 and assessed for convergence in Tracer v.1.6. A graphical representation was made from the combined tree files in FigTree v1.3.1 (Rambaut, 2009).

## 3 RESULTS

We were able to obtain sequences (including partial) from 163 out of 346 samples. Partial sequences, incomplete mainly due to fragmentary ends, were not included in the analyses. Radiocarbon dating was successful for 64 out of 72 brown bear samples, as eight samples did not yield sufficient amounts of purified collagen. Of the successfully amplified samples that produced complete and confirmed sequences (*n* = 136), 28 were previously radiocarbon‐dated and 15 had indirectly inferred dates (Table S1). These samples had an uneven distribution in both time and space. Samples dated to the Holocene dominated (41 of 64), and the Late Pleistocene material (23 of 64) was largely restricted to northwestern Europe (15 of 23). Among the remains recovered from the North Sea for instance, all except one were assigned with ages older than 40,000 cal. B.P. A general trend could also be observed where samples from southern Europe yielded less or no DNA, and there were very few samples reliably dated to the LGM from the Iberian Peninsula or the Balkans. Forty‐five novel haplotypes were identified, of which 40 were represented among the dated samples. In order to include as many published ancient sequences as possible, the alignment was slightly trimmed down to 193 bp.

### 3.1 Genetic relationships of mtDNA haplotypes

**Figure 1:**
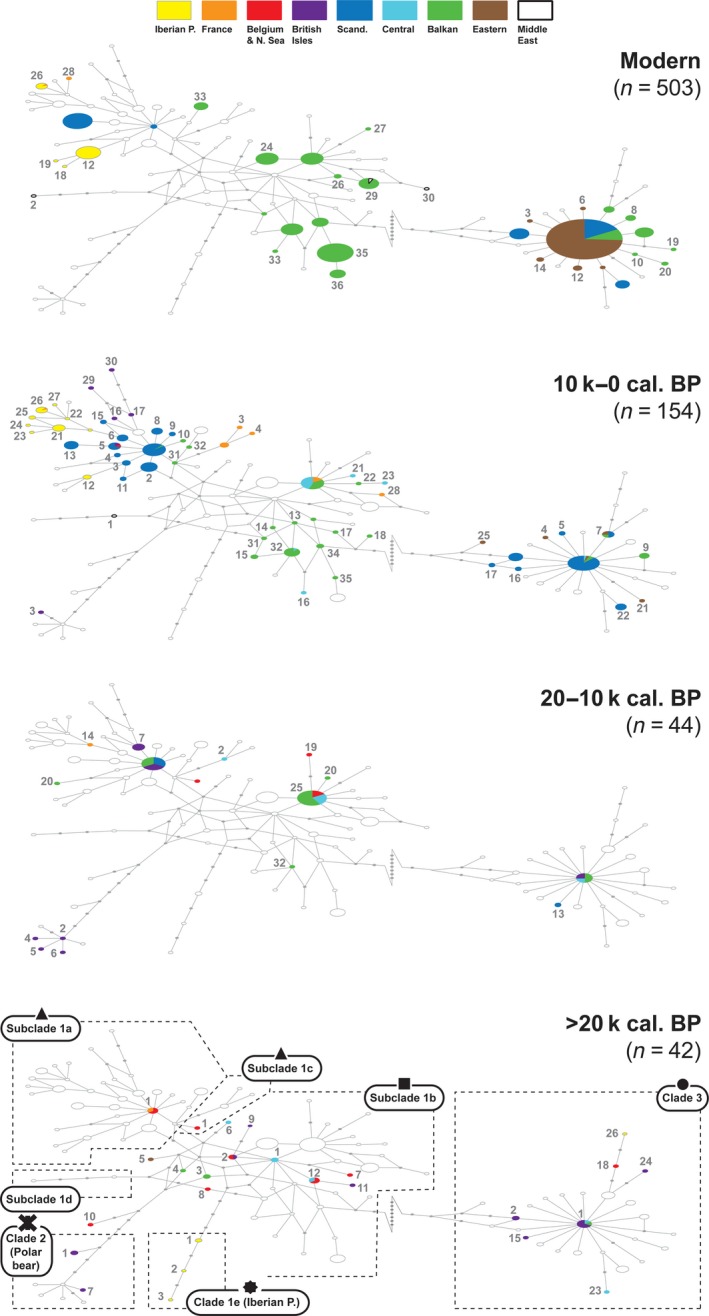
Median‐joining network constructed from the mitochondrial sequences (193 bp) in this study, together with published data of both modern and ancient European brown bears. Samples with unknown age and/or partial sequences were excluded. Numbers indicate haplotypes within the clades. Unbroken connecting lines represent one mutational step, while gray dots indicate intermediate, missing haplotypes

**Figure 2:**
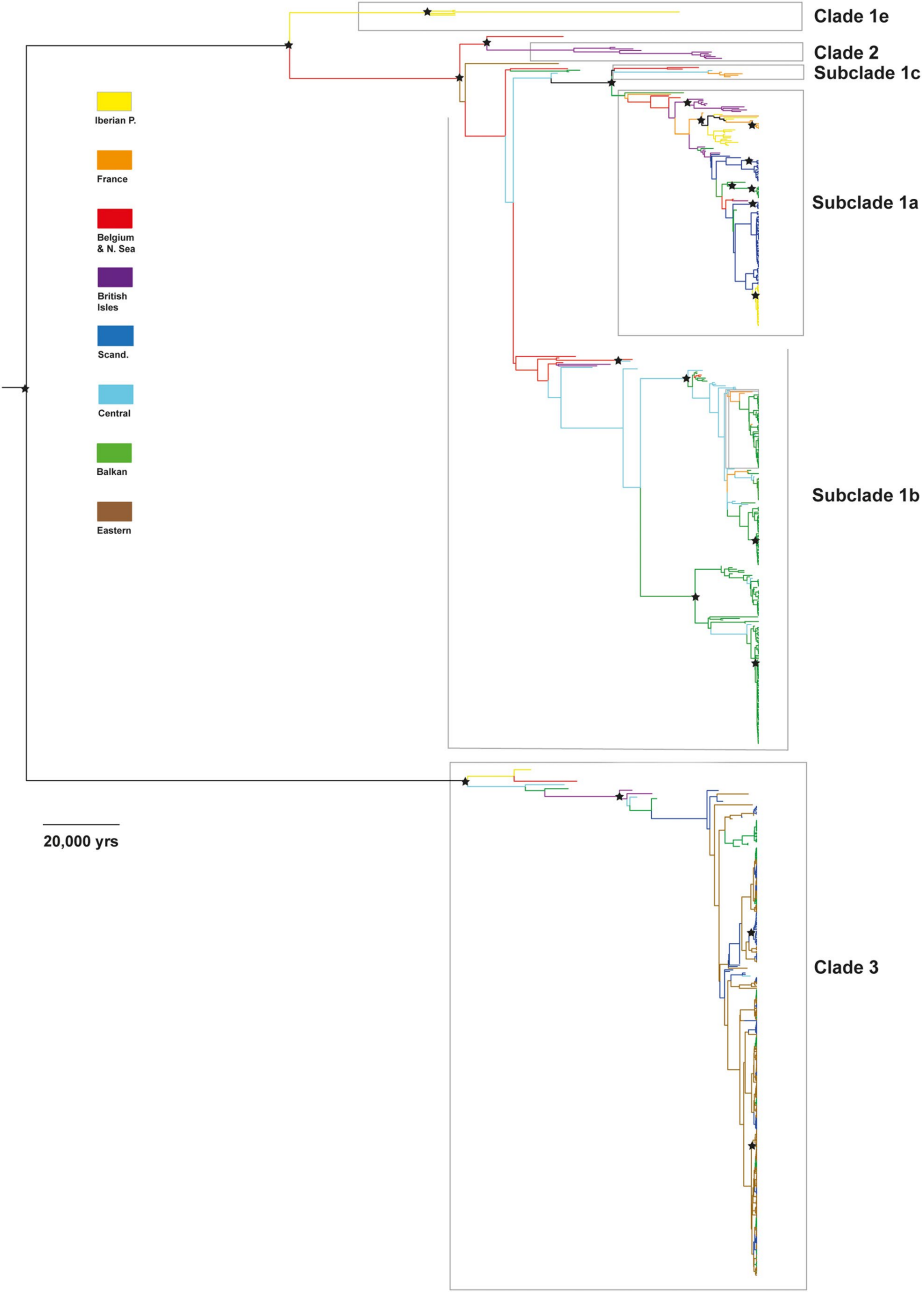
Phylogeny from the BEAST analyses using maximum clade credibility, based on two sets of 100 million trees combined from the posterior distribution. Stars indicate nodes with posterior support above 90%. Branches are colored according to geographic regions. Samples (*n* = 743) were restricted to Europe. Partial sequences and those with unspecified age were excluded

Both the phylogeny and the network analysis showed a clear division between clades 1, 2, and 3 (Figures 1 and 2). A branch representing Late Pleistocene bears from the Iberian Peninsula was also distinct (clade 1e). Although less apparent, there was a division of additional subclades within clade 1. In the network, subclade 1a could be identified based on its noticeable star‐like pattern, with haplotype 1a1, today only found in a Norwegian bear (Taberlet & Bouvet, 1994), as the modal haplotype (Figure 1). Surrounding it was a host of 32 other haplotypes (1a2–1a33), representing bears predominantly from northern and southwestern Europe. A similar modality was also seen in other haplotypes, especially haplotype 1b25, predominantly found in bears from central and eastern Europe. Subclade 1c, first identified in Holocene bears from southern France (Valdiosera et al., 2007), was positioned close to subclade 1a. Although gaining support as a monophyletic branch in the phylogeny, it was difficult to distinguish in the network. One more subclade was outlined in the network: subclade 1d, which was represented by two bears from the Middle East (Calvignac, Hughes, & Hänni, 2009; Çilingir, Akın Pekşen, Ambarlı, Beerli, & Bilgin, 2015).

Interestingly, the new samples belonging to clade 1 deviated from the previous strong geographical pattern seen today in Europe. Bears from the Balkans, especially Bulgaria and the southern Carpathians, were widely represented within clade 1, and several of them even showed a closer affiliation to the western subclade 1a. Moreover, the modal haplotype in the western subclade (haplotype 1a1) was identified in two bears from the southern Carpathians (Ro9 & Ro5), dated to 11,903 and 5,784 cal. B.P., respectively. Most Late Pleistocene samples were found within clade 1, but showed little geographic structure.

clade 3 displayed less diversity and was dominated by the modal haplotype 3.1, which was represented in all time periods. Before the LGM, there was a branch of more divergent haplotypes across western Europe (3.18/24/26), but besides 3.1, almost no haplotypes within this clade showed a continuation through the LGM. Haplotype 3.2 is the only exception, found in bears on the British Isles before the LGM as well as in Scandinavia from historical times.

### 3.2 Range dynamics and recolonization process

**Figure 3:**
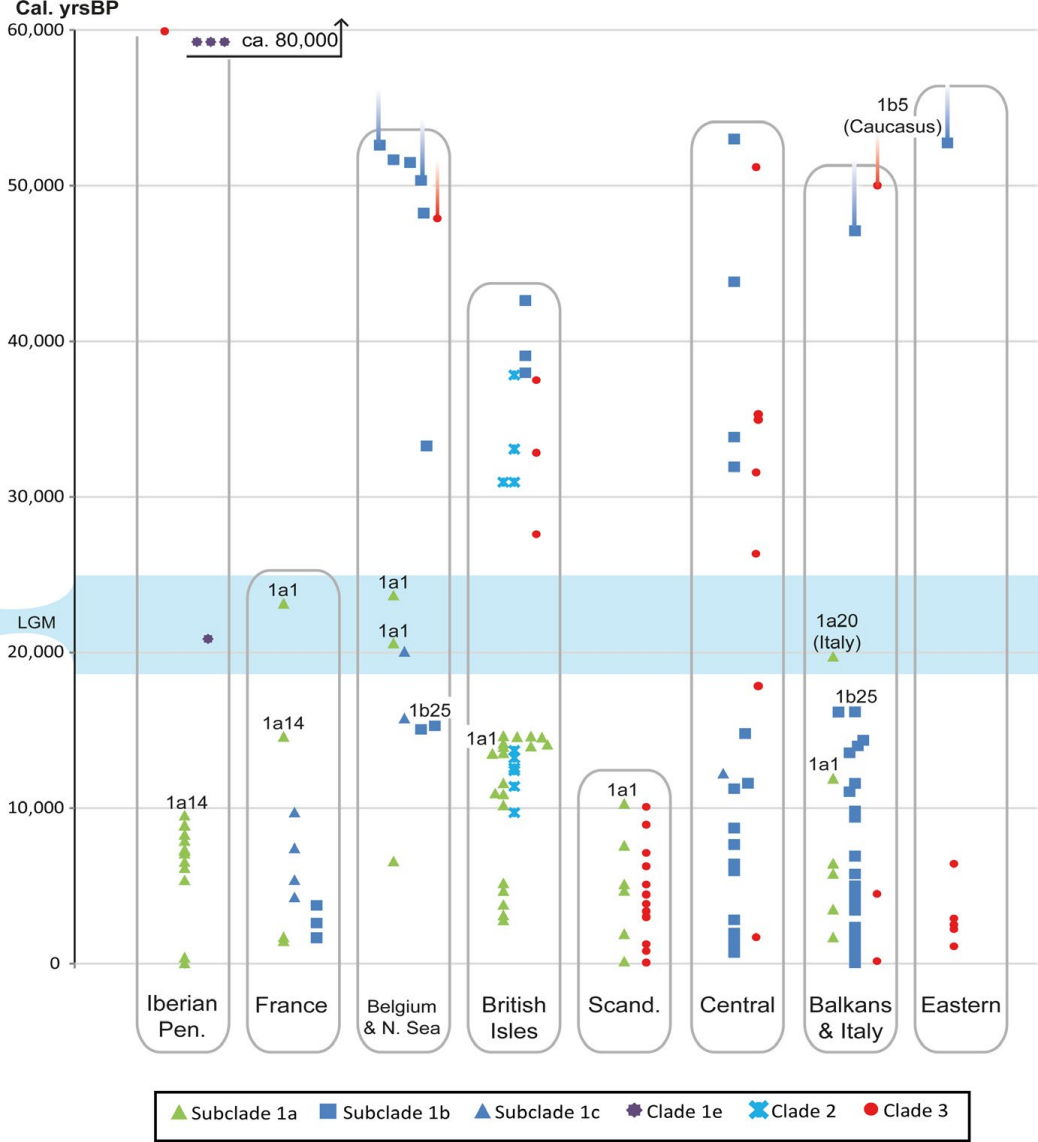
Timeline for the European clades and subclades (1a/b/c, 1e, 2 & 3), divided by geographical regions; Balkans here include Bulgaria, Romania, Slovenia, Hungary, Slovakia, and Poland, central region includes Germany, Switzerland, Austria, Czech Republic, and Denmark, while eastern region includes (European) Russia, Estonia, and Finland. All samples shown are from radiocarbon‐dated remains, with the exception of the four oldest bears from the Iberian Peninsula (at c. 60,000 and 80,000 cal. B.P.). Sequences that were partial but informative of clade were included

**Figure 4:**
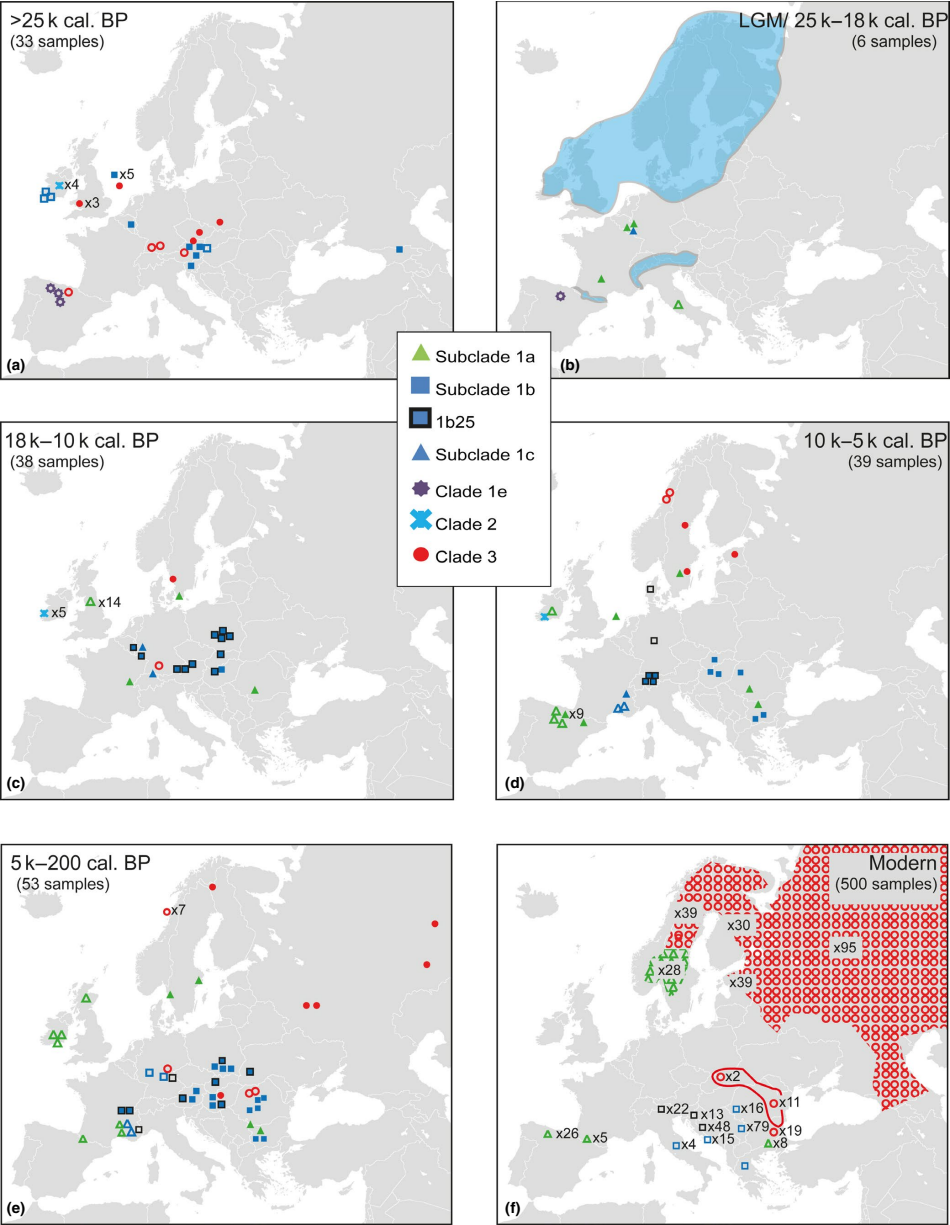
Geographic origin of successfully amplified samples from this study (filled symbols), and previous studies (unfilled symbols), with same restrictions as for Figure 3. Samples are divided into time periods spanning: (a) >25,000 cal. B.P., (b) 25–18,000 cal. B.P. (LGM), (c) 18–10,000 cal. B.P., (d) 10,000–5,000 cal. B.P., (e) 5,000–200 cal. B.P., and (f) Modern times

In contrast to the current eastern distribution of clade 3, bears belonging to this clade were present in central and western Europe before the LGM, including the British Isles (Figures 3 and 4). Even one of the most ancient remains from the North Sea (NS5), dating to beyond c. 50,000 cal. B.P. belonged to clade 3. clade 1b was also represented among the oldest remains, occurring from Ireland in the west to the Caucasus in the east. Both clades 1 and 3 were thus present across Europe with a high degree of genetic variation, but with no apparent geographic structure before the LGM. During this time period, subclade 1a was not found in any of the sampled bears, and bears from the Iberian Peninsula belonged exclusively to clade 3 and the divergent clade 1e (Figures 3 and 4).

During the LGM, a contrasting structure emerged, suggesting that a turnover had taken place. Few samples were represented from this period, but all samples outside the Iberian Peninsula belonged to subclades 1a or 1c (Figures 3 and 4). The oldest bears belonging to subclade 1a were found in Belgium and France, and dated to 23,675 and 23,155 cal. B.P., respectively (Mj7 & Pa19, Figures 3 and 4). At the end of the LGM, an increase in diversity was seen once again (Table 1), with the reappearance of clade 3 and subclade 1b. However, the post‐LGM expansion of subclade 1a was seemingly initiated long before 1b across western Europe, leading the expansion into more peripheral regions. Along with a diversification over time, this spatial expansion of subclade 1a continued into the early Holocene.

**Table 1:** Summary statistics: Measures of genetic diversity across time for the entire European population, including number of haplotypes (HP)

	Europe (all regions)	HP	HP/*n*	HPdiv.	Nuc.div. (π)
Modern	*n* = 500	34	0.066	0.805	0.054
10,000–0 cal. years B.P.	*n* = 153	57	0.37	0.958	0.518
20,000–10,000 cal. years B.P.	*n* = 44	13	0.39	0.853	0.040
>20,000 cal. years B.P.	*n* = 42	26	0.6	0.962	0.060

Note

Abbreviation(s): HPdiv.: haplotype diversity; Nuc.div., nucleotide diversity.

### 3.3 Demographic change

**Figure 5:**
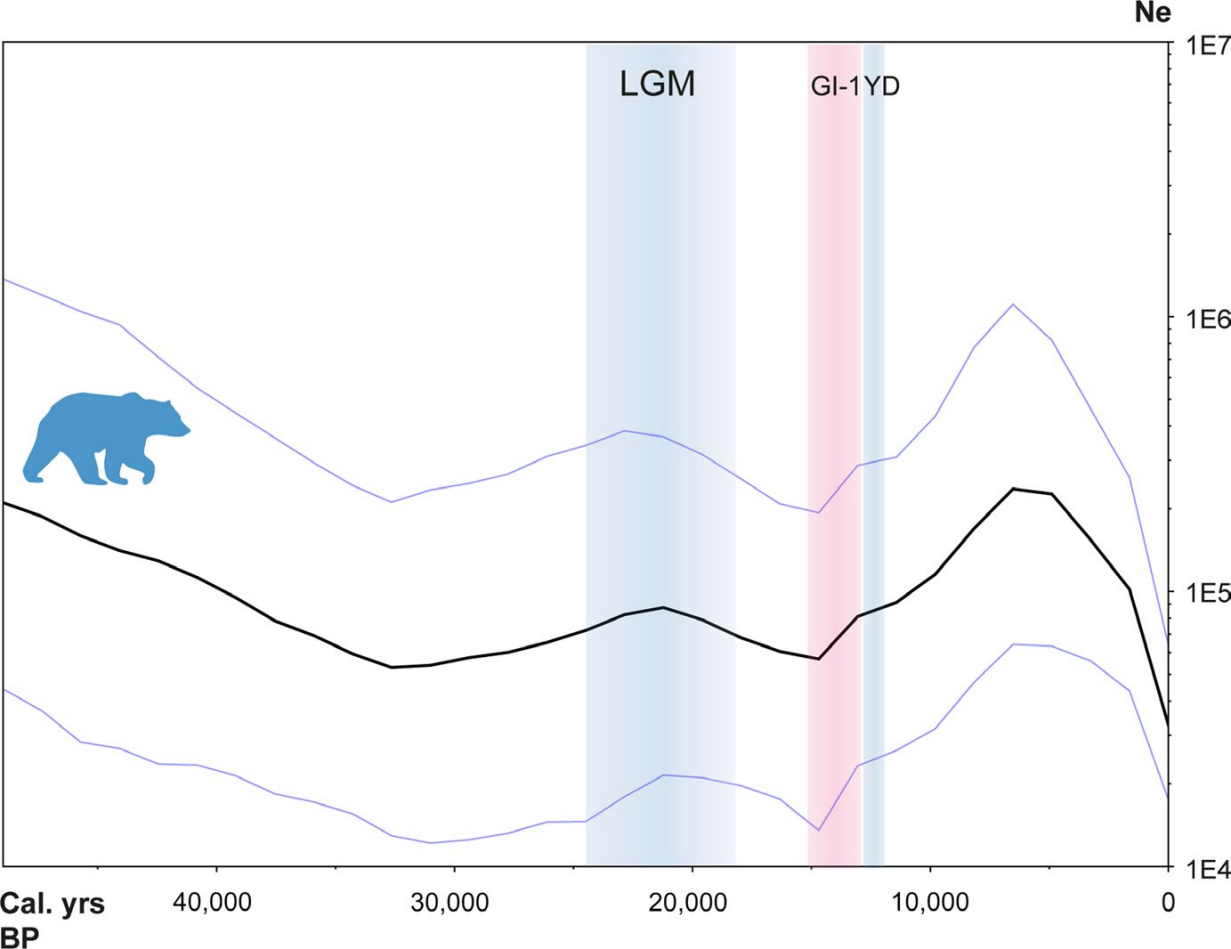
Demographic changes over time: Bayesian Skygrid plot based on dated samples of European brown bears. Solid line plots estimates of the mean effective population size (Ne), with 95% highest posterior density limited by blue lines. Last glacial maximum (LGM) is highlighted in blue as well as the Younger Dryas stadial (YD). Greenland Interstadial 1 (GI‐1) is indicated in pink

A pronounced variation in effective populations size over time was found using the Bayesian Skygrid plot (Figure 5): While a declining trend seemed to prevail in the Late Pleistocene, that is, until c. 30,000 cal. B.P., an increase followed up to the LGM. Then, another decline was initiated during the LGM that continued to c. 15,000 cal. B.P., when a significant increase followed, which lasted into the Holocene. Finally, in the mid‐Holocene, a sharp and almost ten‐fold decline started that lasted up to the present. To explore possible changes within each of the two main clades (1 and 3), the data were accordingly subdivided and analyzed separately. This division does in one way better accommodate a prerequisite of the coalescent models in BEAST, as it is optimally adapted to describe uniform panmictic populations (Drummond, Rambaut, Shapiro, & Pybus, 2005). There was no major difference in the two approaches, that is, similar trends were observed in both the single and the combined analysis.

### 3.4 Stable isotope analysis and dietary changes

**Figure 6:**
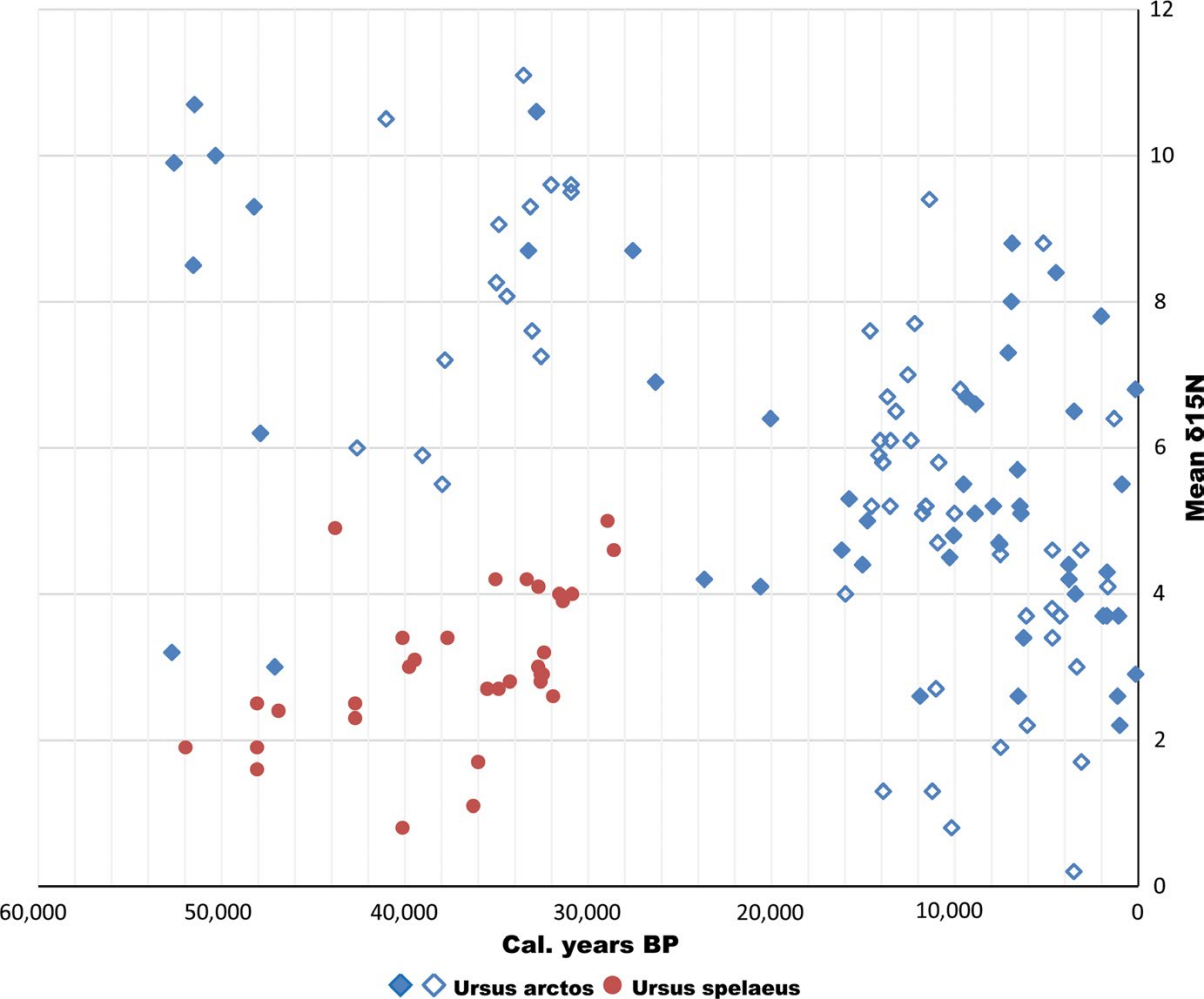
Temporal graph of δ^15^N data from remains of European brown bears and cave bears. Filled diamonds represent samples from this study, while unfilled ones represent already published data. Red dots represent cave bears (Fernández‐Mosquera et al., 2001; Münzel et al., 2011; Stiller et al., 2010)

The great variation in diet that is characteristic for the brown bear was also reflected in our results from the stable carbon and nitrogen isotope analyses. When the isotopic values were analyzed on a temporal scale, a discernible pattern was observed in δ^15^N values, with elevated values in the Late Pleistocene (Figure 6). More specifically, remains radiocarbon dated to before the LGM had significantly higher δ^15^N values compared to samples with more recent radiocarbon dates (Mann–Whitney *U* test, *p* < 0.001). This was also true after a correction for altitude (Table S1), which is known to deflate δ^15^N values (−0.0011 permil/m; Krajcarz et al., 2016; Männel, Auerswald, & Schnyder, 2007). It should be noted that a few of the samples dated to before and during the LGM deviated from this pattern. Two remains from Belgian cave sites dated to the LGM (Mj7 & J11.1) exhibited comparatively low mean δ^15^N values (just over 4 permil), and two older specimens from Slovenia and the Caucasus (Pa2 & G32) had even lower values (3.0 and 3.2 permil).

## 4 DISCUSSION

Despite the addition of samples yielding both DNA and radiocarbon dates in this study, the sampling was still limited. Almost no data were generated from the southern refugia, dated to the LGM or beyond, which prevents conclusions to be made with greater certainty. The relatively short sequences used also placed limits on the phylogenies and haplotypes generated, not least in terms of support for the different clades. As mitochondrial fragments, they only reflect a single locus (the mitochondrial genome) as well as the female lines, which should be considered when interpreting the results.

This study demonstrates a surprisingly complex genetic history of the brown bear in Europe. Contrary to the expectations according to the traditional expansion/contraction model of post‐LGM Europe, we find that brown bears inhabited mid‐latitude Europe throughout the last 50,000 years, including the LGM. Moreover, the finding of several bears in Belgium and France radiocarbon dated to approximately 20,000–23,700 cal. B.P. and carrying the modal haplotype in subclade 1a suggests a scenario where this wide and more northerly region, rather than the Iberian Peninsula, was the origin of the brown bears that later colonized Scandinavia and the British Isles as the ice sheets retreated. Despite a lack of samples from the Iberian Peninsula, it even seems likely that the extant brown bears here might trace their maternal ancestry to bears from this population. The fact that the first haplotype belonging to subclade 1a recorded in the Iberian Peninsula at c. 9,500 cal. B.P. (1a14) was also found in a bear from France around 5,000 years earlier supports this scenario (Figures 2 and 3). An earlier expansion from the Iberian Peninsula might thus have been obstructed by intraspecific competition from brown bears inhabiting France already since the LGM.

Our data also suggest that a genetic turnover took place immediately before the LGM across mid‐latitude Europe, where a mixture of diverse haplotypes of both subclade 1b and clade 3 was largely replaced by a limited set of haplotypes of subclade 1a and 1c. A possible indication of an underlying large‐scale event is the temporal gap between c. 27,600 and 31,600 cal. B.P., which is observed when dated remains from all regions in Europe are jointly compared (Figure 3). Direct causes behind this pattern are difficult to establish, but this time period was notable for several genetic turnovers and extinctions observed in other European species (Cooper et al., 2015). Interestingly, a similar turnover in mitochondrial diversity has been identified for humans in Europe around this time (Posth et al., 2016).

The samples from Belgium and France dated to the LGM confirm that the range of brown bears stretched far to the north across mid‐latitude Europe during this time, something which has only been indirectly implied in previous studies (Valdiosera et al., 2007,2008). This also has implications for hypotheses of glacial refugia at high latitudes (Stewart & Lister, 2001), which have been suggested to have provided suitable habitats for brown bears.

The possibility of glacial survival outside of the traditional southern refugia has also been supported by some recent studies on other European mammals, such as red fox (*Vulpes vulpes*) and red deer (*Cervus elaphus*) (Edwards et al., 2012; Meiri et al., 2013). The introgression of mitochondria from polar bear into Irish brown bears has even led to the idea of a population of brown bears surviving the LGM at this remote location, which has however been subsequently deemed as highly improbable (Leonard et al., 2013). The latter may need to be reviewed due to the data presented here.

The nature and stability of habitats available as far north as Belgium during the LGM are difficult to determine. Two of the radiocarbon‐dated samples from Belgium (Mj7 & J11.1), sharing haplotype 1a, were separated by c. 2,000 years, which indicates a prolonged occurrence of the same population in this region. It is however difficult to estimate population sizes or densities for this population given the limited data at hand. Brown bears are very flexible in terms of habitat choice and it is known that modern bears roam the arctic tundra, but at lower population densities compared to further south (Ferguson & McLoughlin, 2000). It might thus have been possible for a small and scattered population to maintain a presence in Belgium during the LGM, despite harsh conditions. However, an alternative explanation is that the presence of brown bears in Belgium just before 20,000 cal. B.P. represents a short period of significantly warmer climate. It has for instance been shown that two cold‐adapted species, the woolly mammoth (*Mammuthus primigenius*) and the collared lemming (*Dicrostonyx torquatus*), disappeared during short intervals around this time in western Europe (Brace et al., 2012; Stuart, Kosintsev, Higham, & Lister, 2004), which suggests that the northern steppe‐tundra during these periods could have been replaced by habitats capable of sustaining more bears.

Instead of being the result of an expansion from discrete refugia in the south, our results suggest a different process behind the current distinct phylogeographic structure in European brown bears. This process seems to have started with the early expansion of clade 1a in mid‐latitude Europe, which later formed a part of a diverse bear population after the LGM. There are also indications that bears belonging to clade 3 took part in this early expansion. Although a lack of data prevents to establish the origins of these bears, a major refugium for bears belonging to clade 3 has been suggested in the Carpathian Mountains (Saarma et al., 2007). Our results do not rule out this scenario, and clade 3 is indeed present in central Europe already at ca. 17,000 cal. B.P. (Münzel et al., 2011). Also in Scandinavia, the first radiocarbon‐dated remains from the very south belonged to both subclade 1a and clade 3 (Figure 4). The early appearance of clade 3 here suggests that these bears might also have entered the Scandinavian Peninsula from the south, alternatively from the north, along an ice‐free Atlantic coastline.

In subclade 1b, the most notable dynamics start around 16,000 cal. B.P., with an increase in bears carrying haplotype 1b25 (Figures 3 and 4). After this point, 1b25 was widespread across central Europe and was present as far north as Denmark around 6,000 cal. B.P. and in southeastern France at c. 1,700 cal. B.P. Today, this haplotype is also by far the most widespread in the Balkans. However, despite the extensive spread of 1b25, and of subclade 1b in general, it was apparently preceded by subclade 1a in western Europe and there are yet no traces of 1b expanding into the Iberian Peninsula, the British Isles or Scandinavia.

Even though clade 1 is sometimes described as a European clade, recent findings from historical samples have confirmed its presence in bears in the Middle East, which have been grouped in a separate subclade (1d) (Figure 1; Calvignac et al., 2009). In a study on modern Turkish brown bears, both subclade 1b and 1d were represented among three animals (Çilingir et al., 2015), and further to the east, samples around a 100 years old from the Caucasus and western Iran belonged to clade 1 (Hirata et al., 2014). Our results confirm that this eastern distribution also goes far back in time, since one of the oldest samples in this study (G32): a c. 53,000‐year‐old bear from the Caucasus, belonged to clade 1.

During the Holocene, the bear population became increasingly structured and less diverse (Tables 1 and 2), a process which seems to have continued until the present. However, instead of being the result of an expansion from three isolated refugia after the LGM, this appears to have been predominantly a result of decreasing numbers and fragmentation.

**Table 2:** Results from AMOVA test for pairwise population comparison over time

	Western Europe	Φ_ST_	*p*_ΦST_	Eastern Europe
Modern	*n* = 32	0.391	0.001	*n* = 265
10,000–0 cal. years B.P.	*n* = 32	0.288	0.001	*n* = 45
20,000–10,000 cal. years B.P.	*n* = 17	0.266	0.001	*n* = 13
>20,000 cal. years B.P.	*n* = 24	0.00	0.324	*n* = 9

Note

Western Europe included samples from the Iberian Peninsula, France, British Isles, Belgium, and the North Sea. Eastern Europe included central Europe and the Balkans (see Figure 3). Regions further east and Scandinavia were excluded due to limited sample representation over time.

The Skygrid plot (Figure 5) shows that the population of brown bears in Europe suffered considerable changes through time, especially after the LGM. The first increase starting at c. 30,000 cal. B.P. is less intense, but might have been spurred by the extinction of the cave bear, which could have acted as a competitor over habitats in Europe (Münzel et al., 2011). Although the glacial advance during the LGM clearly did not lead to the extinction of bears outside the Mediterranean peninsulas, it certainly may have caused a decrease in available habitat, which might be a cause to the demographic decline starting in the LGM. The subsequent recovery at c. 15,000 cal. B.P. coincides with the onset of Greenland Interstadial 1 (G1‐1), which is characterized by a warming climate followed by a rapid response in vegetation (Williams, Post, Cwynar, Lotter, & Levesque, 2002). This is also more or less synchronous with the first expansion of bears into the British Isles (Edwards et al., 2014; Sommer & Benecke, 2005) as well as the first record of haplotype 1b25 in Hungary (Hu6). Following this, we inferred a steep decline in the brown bear's effective population starting in the mid‐Holocene. This decline coincides with an almost exponential increase in human densities between 8 and 4,000 cal. B.P., based on radiocarbon‐dated samples from archaeological finds in northern and western Europe (Shennan et al., 2013). This indicates that the decline in brown bear abundance during the mid‐Holocene could be linked to the impact of more intense agropastoral land use by humans (Kirby & Watkins, 2015), since this would have led to a loss of suitable bear habitat as well as a decline in wild prey species. It has also been suggested that human changes in husbandry practices may have further excluded prey resources in some regions (Bocherens et al., 2004). Along with the start of this overall decline in the bear population, the first local extinctions are also discerned: Bears belonging to subclade 1c in southeastern France seemingly disappear after c. 4,000 cal. B.P. (Valdiosera et al., 2007). In Denmark, brown bears appear to have become extinct around 4,800–4,400 cal. B.P. (Aaris‐Sørensen, 2009), and in Ireland, the latest radiocarbon‐dated bear has an age of 3,126 cal. B.P. (Edwards et al., 2011). These extinctions are most clearly seen in western Europe, which is also where human‐mediated deforestation first started (Kaplan, Krumhardt, & Zimmermann, 2009). Areas less suitable for agriculture and less accessible to humans seem on the other hand to have retained substantial bear populations. This is also in agreement with a general trend of mountainous areas being less vulnerable to anthropogenic impacts (Faurby & Svenning, 2015). These habitats, often consisting of forested mountains, were more common in the east and served as a kind of Holocene refugium for brown bears. This is also most likely the reason why the most diverse bear population in Europe today is found in the Carpathian Mountains (Straka, Paule, Ionescu, Štofík, & Adamec, 2012).

Although several factors, both environmental and physiological, can influence δ^15^N levels in bears (Bocherens, 2018; Krajcarz et al., 2016), our results from the stable isotope analysis are in line with previous studies, which indicate a significantly higher degree of carnivory in European bears before the LGM (Bocherens, 2015; Münzel et al., 2011). Our data also broaden the temporal range of this pattern with relatively higher δ^15^N values prevailing before 40,000 cal. B.P. (Figure 6). Despite the few data points from the LGM, our results indicate that the shift to lower δ^15^N values started already at around 30,000 cal. B.P. (Figure 6). This is roughly synchronous with the extinction of the cave bear, which has been proposed as the cause behind this dietary shift, as it opened up a more herbivorous niche for brown bears (Bocherens, 2015; Münzel et al., 2011). Another possible reason for the higher δ^15^N values seen before 30,000 cal. B.P. could have been an adaptation to colder and more barren habitats that would have been common in Europe in the Late Pleistocene. It is known that brown bears are generally more carnivorous in open landscapes and at high latitudes (Bojarska & Selva, 2012; Vulla et al., 2009) and that longer hibernation would increase the need for high‐protein food during spring (Fernández‐Mosquera et al., 2001). However, if this was the main factor behind the more carnivorous diet in bears before the LGM, the temporal shift would have been delayed until the very end of the Pleistocene, when the climate became warmer. We thus consider these causative factors to have been less important.

Overall, our study highlights the importance of using ancient DNA for unraveling long‐term processes underlying the phylogeographic structures seen in modern populations (Pääbo, 2000). By combining genetic and isotopic analyses, we have identified previously undetected dynamics in the European brown bear, which points to notable impacts from the changing presence of both cave bears and modern humans. We predict that further analyses, employing DNA techniques targeting nuclear sequences or complete mitochondrial genomes, will enhance the understanding of the dynamics, which we have presented.

## CONFLICT OF INTEREST

None declared.

## AUTHOR CONTRIBUTIONS

LD and EE designed the study and collected samples. EE performed DNA extractions and sequencing, as well as computational analyses, evaluated the results, and drafted the manuscript together with LD. Remaining authors contributed with samples, interpretation of the results, and helped writing the manuscript.

## Data Availability

Novel DNA sequences are available to download from GenBank database (accession numbers MK659705–MK659764).
