# Supplementary material for: Genetic turnovers and northern survival during the last glacial maximum in European brown bears
Source: Ecol Evol. 2019 Apr 16;9(10):5891–905. doi: 10.1002/ece3.5172 (PMC6540696; doi:10.1002/ece3.5172)
Supplement: Supplementary file 1 [file ECE3-9-5891-s001.pdf]

ID	Country	GenBank	Location	Age (^{14}C BP)	Age (cal. BP)	Age (indirect)	HP	(Sub-) Clade	$\delta^{13}C$	$\delta^{15}N$	Alt. (m)
**5SS*[1]**	Spain	MK659718	Sima de Illobi	**8010 ± 45** (OxA-30297)	8885 ± 92		1a25	1a	-19.1	6.6	1185
**8SS*[1]**	Spain	MK659715	Sima de Illobi	**8053 ± 35** (OxA-30243)	8921 ± 91		1a21	1a	-19.6		1185
**12SS*[1]**	Spain		Sima de Illobi	**7063 ± 33** (OxA-30244)	7904 ± 34		1a21	1a	-19.8		1185
**RR1**	Spain		Sima de Illobi	**7069 ± 38** (OxA-30298)	7906 ± 37		1a25	1a	-19.8	5.2	1185
**RR3.3**	Spain	MK659714	Sima de Illobi	**8570 ± 40** (OxA-30296)	9538 ± 14		1a14	1a	-19.7	5.5	1185
**15SS*[1]**	Spain		Vitoria	**7480 ± 50** (X-2586-7)	8293 ± 64		1a21	1a	-19.4		
**17SS*[1]**	Spain	MK659716	Vitoria	**6397 ± 32** (OxA-30245)	7345 ± 52		1a23	1a	-20.0		
**CR1**	Spain	MK659717	Atxagakoa	**6188 ± 35** (OxA-30240)	7091 ± 59		1a24	1a	-18.9	7.3	N/A
**UR.ET. 02**	Spain	MK659719	Cueva del Trocs	**5377 ± 34** (OxA-30299)	6545 ± 55		1a27	1a	-19.5	2.6	N/A
**Aix2**	France		Doubs	**1723 ± 23** (OxA-30246)	1640 ± 45		1b25	1b	-19.8		
**Aix3**	France		Doubs	**2497 ± 26** (OxA-30247)	2605 ± 83		1b25	1b	-20.6		
**Aix4**	France	MK659750	Isère	8740 ± 45	9733 ± 100		1c2	1c			
**Aix5**	France		Doubs	12390 ± 30	14603 ± 327		1a14	1a			
**Sl1**	Slovakia		Belianske Tatry Mts	**456 ± 25** (OxA-30248)	515 ± 8		1b25	1b	-20.8		1548
**Sl2**	Slovakia	MK659733	Západné Tatry Mts.	**5003 ± 30** (OxA-30249)	5766 ± 80		1b14	1b			1133
**Sl3**	Slovakia	MK659743	Nizke Tatry Mts.	**11665 ± 50** (OxA-30250)	13551 ± 134		1b25	1b			900
**G3**	Russia	MK659760	Kama River			ca. 1100	3.21	3			
**G6**	Russia	MK659753	Urals			2300-2700	3.4	3			
**G7**	Russia	MK659754	European Russia			2800-3000	3.7	3			
**G9**	Estonia	MK659751	Unknown	**5628 ± 33** (OxA-30300)	6400 ± 47		3.1	3	-21.2	5.1	N/A
**G15**	Russia	MK659763	European Russia			ca 2200	3.25	3			
**G20**	Ukraine		Luka Vrobletski			ca. 4000	1b25	1b			
**G32**	Georgia	MK659726	Kudaro 1 Cave, layer 3, Caucasus	**>48600** (OxA-32245)	(>52722)		1b5	1b	-19.6	3.2	1600
**Ro2**	Romania	MK659745	Fagaras mts.	**372 ± 24** (OxA-30257)	416 ± 67		1b32	1b			
**Ro3**	Romania	MK659746	Mehedinti mts.	**1109 ± 26** (OxA-30214)	1017 ± 32		1b34	1b	-20.5	2.2	N/A
**Ro4**	Romania		Mehedinti mts.	**1160 ± 25** (OxA-30215)	1078 ± 55		1b32	1b	-19.9	3.7	N/A

**Ro5**	Romania	MK659705	Mehedinti mts.	**5018 ± 34** (OxA-30259)	5784 ± 77		1a1	1a	-18.6		
**Ro6**	Romania	MK659731	Mehedinti mts.	**1900 AD** (OxA-30260)			1b12	1b	-20.8		
**Ro8**	Romania		Southern Carpathians	**188 ± 23** (OxA-30261)	153 ± 125		3.7	3	-20.0		
**Ro9**	Romania		Southern Carpathians	**10205 ± 55** (OxA-30295)	11903 ± 147		1a1	1a	-19.3	2.6	N/A
**Ro10**	Romania		Fagaras mts.	**125 ± 25** (OxA-30258)	139 ± 99		1b34	1b	-18.5		
**H1**	Romania	MK659741	Apuseni mts.	**988 ± 24** (OxA-30134)	891 ± 24		1b22	1b	-19.5	5.5	N/A
**Hu6**	Hungary		Szelim-barlang, B réteg, Komárom megye	**13255 ± 60** (OxA-30262)	16185 ± 409		1b25	1b	-19.5		
**Hu9**	Hungary	MK659739	Szelim-barlang, B réteg, Komárom megye	**13240 ± 28** (OxA-30268)	16170 ± 399		1b20	1b	-19.8	4.6	292
**Hu10**	Hungary		Varbó, Lambrecht Kálmán-barlang	**6064 ± 32** (OxA-30699)	6925 ± 40		1b32	1b	-18.8	8.0	410
**Hu11**	Hungary		Varbó, Lambrecht Kálmán-barlang	**6038 ± 33** (OxA-30237)	6886 ± 50		1b32	1b	-18.0	8.8	410
**Hu12**	Hungary		Varbó, Lambrecht Kálmán-barlang, P/II. réteg	**2052 ± 25** (OxA-30263)	2022 ± 36		1b25	1b	-18.9	7.8	410
**Hu15**	Hungary	MK659734	Varbó, Lambrecht Kálmán-barlang, P/II. réteg	**3499 ± 28** (OxA-30238)	3778 ± 45		1b15	1b	-20.0	4.2	410
**Hu18**	Hungary		Varbó, Lambrecht Kálmán-barlang, P/I. réteg	**3528 ± 30** (OxA-30213)	3801 ± 54		1b15	1b	-19.4	4.4	410
**Hu20**	Hungary	MK659755	Peskő-barlang, humusz	**4017 ± 31** (OxA-30239)	4484 ± 36		3.9	3	-18.8	8.4	745
**Bu1**	Bulgaria	MK659737	Dolnoslav			ca. 5000	1b18	1b			
**Bu3**	Bulgaria	MK659736	Pirin mountain	**8367 ± 39** (OxA-30270)	9391 ± 58		1b17	1b	-18.7	6.7	N/A
**Bu6**	Bulgaria	MK659747	Temnata dupka	**180 ± 22** (OxA-30562)	149 ± 125		1b35	1b	-17.1	2.9	420
**Bu8**	Bulgaria	MK659721	Svinskata cave	**1783 ± 27** (OxA-30403)	1709 ± 59		1a32	1a	-19.2	4.3	450
**Bu9**	Bulgaria	MK659713	Baley, N-W Bulgaria	**3267 ± 27** (OxA-30133)	3505 ± 41		1a10	1a	-19.8	6.5	100
**Bu12**	Bulgaria	MK659744	Mazeta/Mazeto cave	**3216 ± 31** (OxA-30419)	3434 ± 28		1b31	1b	-18.5	4.0	1350

**Bu15**	Bulgaria	MK659732	District of Trun			ca. 200	1b13	1b			
**Bu16**	Bulgaria	MK659720	Unknown loc./ P4,4	**5665 ± 50** (OxA-32244)	6456 ± 53		1a31	1a	-19.6	5.2	N/A
**Pa2**	Slovenia	MK659724	Matjazeve kamer	**>43500** (Beta-422073)	(>47102)		1b3	1b	-19.0	3.0	540
**Pa9**	Czech Rep.		Pod hradem	**21940 ± 140** (OxA-30136)	26330 ± 382		3.1	3	-19.5	6.9	N/A
**Pa10**	Austria		Gudenus cave	**12480 ± 55** (OxA-30264)	14784 ± 299		1b25	1b	-19.5	5.0	496
**Pa11**	Austria		Krems-Wachtberg	26800 ± 220	31561 ± 251		3.1	3			260
**Pa13**	Austria	MK659727	Herdengel-cave	53000 ± 1700	53000		1b6	1b			878
**Pa14**	Austria	MK659722	Repolust-cave	27270±220	31930 ± 179		1b1	1b			525
**Pa19**	France		Igue du Gral	19390 ± 100	23155 ± 283		1a1	1a			
**Pa20*[2]**	Switzerland		Geissbachhöhle	6810 ± 50	7650 ± 36		1b25	1b			1260
**Pa21*[3]**	Austria	MK659740	Wildes Loch	1210 ± 30	1137 ± 45		1b21	1b	-20.1	2.6	1796
**Pa22*[3]**	Austria		Laufenbergloch	9810 ± 70	11241 ± 51		1b25	1b	-20.3	1.3	1445
**Pa25*[2]**	Austria		Bärenfalle	1730 +-30	1643 ± 46		1b25	1b			
**Pa26*[3]**	Germany		Schlüssellochhöhle	10055 ± 33	11585 ± 149		1b25	1b	-18.7	5.2	1275
**Pa27*[3]**	Switzerland		Bärenhöhle am Stoss	5260 ± 65	6060 ± 96		1b25	1b	-19.8	2.2	1165
**Pa29*[2]**	Switzerland		Bärenfalle, Silberen	7845 ± 75	8707 ± 136		1b25	1b			2070
**Pa30**	Switzerland		Neuschneehöhle	5610 ± 70	6403 ± 69		1b25	1b			1680
**Pa31**	Austria	MK659742	Eichberghöhle	2695 ± 45	2810 ± 37		1b23	1b			850
**Pa32**	Romania		Pestera cu Ceata	8790 ± 35	9813 ± 74		1b32	1b			
**Pa34*[2]**	Switzerland		Wildenmannlisloch	10325 ± 50	12221 ± 171		1c2	1c			1620
**M1**	Austria		Mariannenhöhle	29475 ±174	33840 ± 302		1b12	1b			636
**Mj4**	Belgium	MK659738	Furfooz			Post-LGM	1b19	1b			
**Mj5**	Belgium	MK659749	Goyet, 3e caverne	**12960 ± 65** (OxA-32248)	15783 ± 405		1c1	1c	-19.8	5.3	130
**Mj6**	Belgium		Goyet, 3e caverne	**28800 ± 320** (OxA-32250)	33277 ± 461		1b12	1b	-19.2	8.7	130
**Mj7**	Belgium		Goyet, 3e caverne	**19770 ± 130** (OxA-32251)	23675 ± 298		1a1	1a	-19.8	4.2	130
**Mj8**	Belgium		Goyet, 3e caverne	**16840 ± 90** (OxA-32252)	20063 ± 280		1c1	1c	-19.3	6.4	130
**J1**	Belgium		Trou al'Wesse	**12670 ± 60** (OxA-30404)	15047 ± 307		1b25	1b	-19.8	4.4	200

**J2**	Belgium		Trou al'Wesse	**12800 ± 60** (OxA-30251)	15285 ± 256		1b25	1b	-20.0		
**J11.1**	Belgium		Trou al'Wesse	**17170 ± 90** (OxA-32186)	20609 ± 303		1a1	1a	-19.3	4.1	200
**J16**	UK (Wales)	MK659730	Little Hoyle			LGM?	1b11	1b			
**J17**	UK (Wales)		Little Hoyle	**23010 ± 180** (OxA-32246)	27579 ± 450		3.1	3	-17.7	8.7	<100
**J20**	UK (Wales)	MK659752	Pontnewydd Cave			Pre-LGM	3.2	3			
**J23**	UK (Wales)		Pontnewydd Cave			Pre-LGM	3.2	3			
**J26**	UK (Wales)	MK659757	Pontnewydd Cave			Pre-LGM	3.15	3			
**J27**	UK (Wales)		Pontnewydd Cave	32900 ± 800	37490 ± 1175		3.1	3			
**J29**	UK (Wales)		Pontnewydd Cave			Pre-LGM	3.1	3			
**J30**	UK (Wales)		Pontnewydd Cave	**28400 ± 300** (OxA-32247)	32829 ± 423		3.1	3	-19.6	10.6	<100
**J32**	UK (Wales)		Pontnewydd Cave			Pre-LGM	3.1	3			
**J33**	UK (Wales)	MK659762	Pontnewydd Cave			Pre-LGM	3.24	3			
**J34**	UK (Wales)	MK659710	Penwyllt Cave			Late Glacial	1a7	1a			
**J35**	UK (Wales)		Penwyllt Cave			Late Glacial	1a1	1a			
**J36**	UK (Wales)		Priory Farm Cave			Late Glacial	3.1	3			
**R5**	Netherlands	MK659723	North Sea	**47100 ± 2900** (OxA-32256)	51555 ± 4035		1b2	1b	-19.7	8.5	<100
**NS2**	Netherlands	MK659707	North Sea	**47100 ± 2800** (OxA-30157)	51489 ± 3925		1(b)10	?	-19.4	10.7	<100
**NS3**	Netherlands	MK659709	North Sea	**5781 ± 33** (OxA-30158)	6586 ± 48		1a5	1a	-21.3	5.7	<100
**NS5**	Netherlands	MK659761	North Sea	**>44500** (OxA-30159)	(>47895)		3.18	3	-19.5	6.2	<100
**NS17**	Netherlands		North Sea	**>48500** (OxA-32253)	(>52598)		1b12	1b	-19.8	9.9	<100
**NS20**	Netherlands	MK659729	North Sea	**>46800** (OxA-32254)	(>50333)		1b8	1b	-19.8	10.0	<100
**NS23**	Netherlands	MK659728	North Sea	**44500 ± 2100** (OxA-32255)	48237 ± 2544		1b7	1b	-19.5	9.3	<100
**Mz4**	Germany	MK659735	Mainz			ca 1950	1b16	1b			
**Pl1**	Poland		Ruska Skała Rock Shelter	3935 ± 35	4545 ± 99		1b32	1b			
**Pl2**	Poland	MK659725	Północna Duża Cave			14-29 kBP	1b4	1b			
**Pl3**	Poland		Południowa Cave	9860 ± 50	11492 ± 98		1b25	1b			
**Pl4**	Poland		Niedźwiedzia Cave			<14000	1b25	1b			
**Pl7**	Poland		Wschodnia Cave	12170 ± 70	14206 ± 236		1b25	1b			
**Pl9**	Poland		Biśnik Cave	>50000	>50000		3.1	3			

**Pl11**	Poland		Sokole Mts			<14000	3.1	3			
**Pl16**	Poland		Deszczowa Cave	4000 ± 100	4500±170		1b25	1b			
**Pl17**	Poland		Dziadowa Skała Cave			ca 12000	1b25	1b			
**Pl18**	Poland		Grota Kasprowa, Tatra Mts.			ca 50	1b32	1b			
**Pl19*[4]**	Poland		Komarowa Cave	12260 ± 60	14346 ± 276		1b25	1b			
**Pl22**	Poland		Cave in Tatra Mts.	2330 ± 30	2190 ± 30		1b32	1b			
**Pl23**	Poland		Nad Zagonem Cave (Zielona Cave), Tatra Mts.			ca 1800	1b32	1b			
**Sw1**	Sweden	MK659708	Motala, kanaljorden			ca 7600	1a4	1a	-20.5	4.7	<100
**Sw4**	Sweden		Smedstorp, Skåne	**9105 ± 50** (OxA-32192)	10290 ± 57		1a1	1a	-20.7	4.5	<100
**Sw8**	Finland	MK659764	Enontek iö näkkälä	830 ± 25	740 ± 27		3.22	3			
**Sw9**	Sweden	MK659711	Sotenäs kanal	**1959 ± 27** (OxA-32193)	1916 ± 27		1a8	1a	-20.6	3.7	<100
**Sw11**	Sweden	MK659759	Sturefors, Östergötland	**8040 ± 30** (Beta-419016)	8913 ± 92		3.17	3	-19.9	5.1	<100
**Sw12**	Sweden	MK659756	Årnarp, Halland	**8955 ± 45** (OxA-32193)	10081 ± 107		3.13	3	-20.7	4.8	<100
**Sw13**	Sweden	MK659758	Erikslund, Sillre, Medelpad	**5443 ± 30** (X-2581-54)	6254 ± 31		3.16	3	-20.2	3.4	<100
**Sw19**	Sweden	MK659712	Grödinge/Korsnäs	4145 ± 40	4699 ± 90		1a9	1a			
**GX22**	Norway	MK659706	Skien	**192 ± 26** (OxA-32236)	156 ± 124		1a2	1a	-19.6	6.8	<100
**GX47**	Norway		Karlebotn		(1880 AD)		3.22	3			
**Sw37**	Norway		Alten		(1894 AD)		3.22	3			
**J8**	Belgium		Trou al Wesse			LP-Early Holocene	Partial	1b			
**NS1**	Netherlands		North Sea			LP-Early Holocene	Partial	1b			
**NS9**	Netherlands		North Sea			LP-Early Holocene	Partial	1b			
**NS13**	Netherlands		North Sea			LP-Early Holocene	Partial	1b			
**NS19**	Netherlands		North Sea			LP-Early Holocene	Partial	3			
**NS32**	Netherlands		North Sea			LP-Early Holocene	Partial	1a			
**Mz1**	Germany		Gimbsheim			LP-Early Holocene	Partial	1b			
**Sw20**	Sweden		Alvastra pålbyggnad			ca 5100	Partial	1a			
**Bc1**	Spain		La Garrotxa			Late Pleistocene	No DNA				
**Bc2**	Spain		La Garrotxa			Late Pleistocene	No DNA				

**Bc3**	Spain	La Garrotxa	Late Pleistocene	No DNA		
**Bc4**	Spain	Ullastret, Girona	Late Pleistocene	No DNA		
**Bc5**	Spain	Santa Pau de Gaudixa	Late Pleistocene	No DNA		
**Bc6**	Spain	Cova dels Muriceus (Lleida)	Late Pleistocene	No DNA		
**Bc7**	Spain	Cova dels Muriceus (Lleida)	Late Pleistocene	No DNA		
**Bc8**	Spain	Cova dels Muriceus (Lleida)	Late Pleistocene	No DNA		
**Bc9**	Spain	Cova dels Muriceus (Lleida)	Late Pleistocene	No DNA		
**Bc10**	Spain	Cova dels Muriceus (Lleida)	Late Pleistocene	No DNA		
**Bc11**	Spain	Cova dels Muriceus (Lleida)	Late Pleistocene	No DNA		
**Bc12**	Spain	Cova dels Muriceus (Lleida)	Late Pleistocene	No DNA		
**1SS*[1]**	Spain	Sima de Illobi	Pleistocene/Holocene	No DNA		1185
**2SS*[1]**	Spain	Sima de Illobi	Pleistocene/Holocene	1a21	1a	1185
**3SS*[1]**	Spain	Sima de Illobi	Pleistocene/Holocene	1a21	1a	1185
**4SS*[1]**	Spain	Sima de Illobi	Pleistocene/Holocene	1a21	1a	1185
**6SS*[1]**	Spain	Sima de Illobi	Pleistocene/Holocene	1a21	1a	1185
**7SS*[1]**	Spain	Sima de Illobi	Pleistocene/Holocene	1a21	1a	1185
**9SS*[1]**	Spain	Sima de Illobi	Pleistocene/Holocene	1a21	1a	1185
**10SS*[1]**	Spain	Sima de Illobi	Pleistocene/Holocene	1a25	1a	1185
**11SS*[1]**	Spain	Sima de Illobi	Pleistocene/Holocene	1a21	1a	1185
**13SS*[1]**	Spain	Vitoria region	Pleistocene/Holocene	1a21	1a	
**14SS*[1]**	Spain	Vitoria region	Pleistocene/Holocene	No DNA		
**16SS*[1]**	Spain	Vitoria region	Pleistocene/Holocene	No DNA		
**18SS*[1]**	Spain	Vitoria region	Pleistocene/Holocene	No DNA		
**19SS*[1]**	Spain	Vitoria region	Pleistocene/Holocene	No DNA		
**Cr1**	Croatia	Krapina	Late Pleistocene	No DNA		
**Cr2**	Croatia	Krapina	Late Pleistocene	No DNA		
**Cr3**	Croatia	Krapina	Late Pleistocene	No DNA		
**Cr4**	Croatia	Krapina	Late Pleistocene	No DNA		
**Aix1**	France	Doubs	Holocene?	1b25	1b	

**Sl4**	Slovakia	MK659748	Jaskyňa Izabely Textorisovej Cave, Velká Fatra Mts.		?	1b37	1b	754
**Sl5**	Slovakia		Trencianske Bohuslavice		LGM	No DNA		210
**Sl6**	Slovakia		Trencianske Bohuslavice		LGM	No DNA		210
**Sl7**	Slovakia		Trencianske Bohuslavice		LGM	No DNA		210
**Sl8**	Slovakia		Trencianske Bohuslavice		LGM	No DNA		210
**Sl9**	Slovakia		Trencianske Bohuslavice		LGM	No DNA		210
**Sl10**	Slovakia		Trencianske Bohuslavice		LGM	No DNA		210
**Sl11**	Slovakia		Trencianske Bohuslavice		LGM	No DNA		210
**G2**	Russia		European Russia		?	No DNA		
**G10**	Russia		Eliseevichi, russian plains		Late Pleistocene	No DNA		
**G13**	Russia		Urals		Late Pleistocene	Partial	3	
**G14**	Uzbekistan		Aman Koutan Cave		Mousterian (LP)	No DNA		
**G15**	Russia		European Russia?		2200	No DNA		
**G16**	Russia		Volga River, SW Russia		Middle Pleistocene?	No DNA		
**G17**	Russia		Shan-Koba, Crimea		Early Holocene?	No DNA		
**G21**	Moldova		Unknown		Bronze age?	No DNA		
**G24**	Russia		Tver Region (Eur)		Neolithic (late)	No DNA		
**G25**	Russia		Tver Region (Eur)		Neolithic (late)	No DNA		
**G26**	Russia		Tver Region (Eur)		Neolithic (late)	No DNA		
**G27**	Russia		Tver Region (Eur)		Neolithic (late)	No DNA		
**G29**	Georgia		Kudaro 1 Cave, Caucasus		Late Pleistocene	No DNA		
**G30**	Georgia		Kudaro 1 Cave, layer 2, Caucasus	**>49900** (OxA-30256)		U. kudarensis		
**G31**	Georgia		Kudaro 3 Cave, layer 3c, Caucasus		Late Pleistocene	No DNA		
**G33**	Georgia		Kudaro 1 Cave, layer X, Caucasus		Late Pleistocene	No DNA		
**Ro1**	Romania		Casian		MIS 3?	No DNA		
**Ro7**	Romania		Eastern Carpathians		?	No DNA		
**Ro11**	Romania		Muerilor cave	**33750 ± 550** (OxA-30560)		U. spelaeus		
**It1**	Italy		Columbare, Negrar Verona		Holocene site	Partial	1c	
**It2**	Italy		Veje A I shats Verona		?	No DNA		

**It3**	Italy	Lecce Sole, Falciano Selice	?	No DNA	
**It4**	Italy	Lecce Sole, Falciano Selice	?	No DNA	
**It5**	Italy	Lecce Sole, Falciano Selice	?	No DNA	
**It6**	Italy	Fumane di Corredo	Bronze age	No DNA	
**It7**	Italy	Cerè cave sant´anna d´Alfredo VR	Pleistocene (Riss)	No DNA	
**It8**	Italy	Cerè cave sant´anna d´Alfredo VR	Pleistocene (Riss)	No DNA	
**It9**	Italy	Banche di Sofferino, Montova	Bronze age	No DNA	
**It10**	Italy	Sealucca - Molina (Verona)	?	No DNA	
**It11**	Italy	Avaro (Riparo Mezzena) Verona	Pleistocene site	No DNA	
**It12**	Italy	Avaro (Riparo Zampieri) Verona	Pleistocene site	No DNA	
**It13**	Italy	Falciano Caserta (Campania)	Pleistocene site	No DNA	
**It14**	Italy	Ferrara di Monte Baldo VR	Pleistocene site	No DNA	
**It15**	Italy	Ferrara di Monte Baldo VR	Pleistocene site	No DNA	
**It16**	Italy	Ferrara di Monte Baldo VR	Pleistocene site	No DNA	
**It17**	Italy	Soave (Verona)	Pleistocene site	No DNA	
**Hu1**	Hungary	Szelim-barlang	Late Pleistocene	No DNA	
**Hu2**	Hungary	Szelim-barlang	Late Pleistocene	No DNA	
**Hu3**	Hungary	Szelim-barlang	Late Pleistocene	1b25	1b
**Hu4**	Hungary	Szelim-barlang	Late Pleistocene	No DNA	
**Hu5**	Hungary	Szelim-barlang	Late Pleistocene	No DNA	
**Hu7**	Hungary	Szelim-barlang	Late Pleistocene	No DNA	
**Hu8**	Hungary	Szelim-barlang	Late Pleistocene	1b25	1b
**Hu13**	Hungary	Lambrecht Kálmán-barlang	?	Partial	1b
**Hu14**	Hungary	Lambrecht Kálmán-barlang	?	No DNA	
**Hu16**	Hungary	Lambrecht Kálmán-barlang	?	No DNA	
**Hu17**	Hungary	Lambrecht Kálmán-barlang	?	No DNA	
**Hu19**	Hungary	Lambrecht Kálmán-barlang	?	No DNA	
**Hu21**	Hungary	Ölyveskő	?	No DNA	
**Hu22**	Hungary	Tarkői-fülke	?	No DNA	
**Hu23**	Hungary	Tarkői-fülke	?	No DNA	

**Hu24**	Hungary	Tarkői-fülke	?	No DNA	
**Hu25**	Hungary	Istállóskői-barlang	Late Pleistocene	No DNA	
**Hu26**	Hungary	Zalaegerszeg, 2. téglagyár	Late Pleistocene	No DNA	
**Hu27**	Hungary	Vesszőstetői-barlang, Bükk	Late Pleistocene	No DNA	
**Hu28**	Hungary	Süttő, Kőbánya	Late Pleistocene	No DNA	
**Hu29**	Hungary	Diósgyőri-barlang	Late Pleistocene	No DNA	
**Hu30**	Hungary	Alsó-Szinva-forrás feletti barlang	Late Pleistocene	No DNA	
**Hu31**	Hungary	Szuhogy	Late Pleistocene	Partial	1b
**Hu32**	Hungary	Sikaliktya, Keszthelyi-hegység	Holocene	No DNA	
**Hu33**	Hungary	Porlyuk-barlang	Late Pleistocene	No DNA	
**Hu34**	Hungary	Porlyuk-barlang	Late Pleistocene	No DNA	
**Hu35**	Hungary	Tarkő, III.	Late Pleistocene	No DNA	
**Hu36**	Hungary	Tarkő, IV.	Late Pleistocene	No DNA	
**Hu37**	Hungary	Tarkő, IV.	Late Pleistocene	No DNA	
**Hu38**	Hungary	Tarkő, IV.	Late Pleistocene	No DNA	
**Hu40**	Hungary	Tarkő, "A" réteg	Late Pleistocene	No DNA	
**Bu2**	Bulgaria	Dolnoslav. SM 1854	?	1b16	1b
**Bu4**	Bulgaria	992, Teteven	Pleistocene	No DNA	
**Bu7**	Bulgaria	Svinskata cave	Late Pleistocene	No DNA	
**Bu10**	Bulgaria	Baley, N-W Bulgaria	Early bronze age	No DNA	
**Bu11**	Bulgaria	Baley, N-W Bulgaria	Early bronze age	No DNA	
**Bu13**	Bulgaria	Gradeshnitsa	Early neolithic	No DNA	
**Bu14**	Bulgaria	Gradeshnitsa	Early neolithic	No DNA	
**Bu17**	Bulgaria	Unknown loc./ D/15	Prehistory	No DNA	
**Bu18**	Bulgaria	Unknown loc./8-x	Prehistory	No DNA	
**Bu19**	Bulgaria	Unknown loc./D/15	Prehistory	No DNA	
**Bu20**	Bulgaria	Unknown loc./D/7	Prehistory	No DNA	
**Pa1**	Czech Rep.	Pavlov	?	No DNA	
**Pa3**	Slovenia	Ajdovska jama	?	No DNA	
**Pa4**	Russia	Krasni Yar	?	No DNA	

**Pa5**	Russia	Mahnevskaya		?	No DNA		
**Pa6**	Austria	Schusterlucke	**25870 ± 210** (OxA-30135)		U. spelaeus		
**Pa7**	Russia	Dinamitnaya		?	No DNA		
**Pa8**	?	Barsucha cave		?	No DNA		
**Pa12**	Czech Rep.	Predmost	25350 ± 130		No DNA		
**Pa15**	Russia	Nizhny Tavda	>48700		No DNA		
**Pa16**	Russia	Krasni Yar	47500 ± 900		No DNA		
**Pa17**	Russia	Apatskoe	>50900		No DNA		
**Pa18**	Romania	Onceasa	20980 ± 110		No DNA		
**Pa24**	Austria	Bärenhöhle im Stockerstein		?	1b25	1b	
**Pa28**	Switzerland	Milchbalmhöhle	9690 ± 75		No DNA		1622
**Pa33**	Turkey	Cukurici Höyuk		Bronze Age	No DNA		
**Pa35**	Austria	Gamssulzencave		?	No DNA		1300
**Pa37**	Austria	Josefinengrotte		Pleistocene?	No DNA		
**Pa38**	Austria	Badlhöhle		Pleistocene?	No DNA		495
**Pa39**	Austria	Kugelstein II		Pleistocene?	No DNA		485
**Mj2**	Belgium	Trou de Chaleux		Post-LGM	1b25	1b	
**Mj3**	Belgium	Furfooz, Trou des Nutons		Post-LGM	No DNA		
**Mj9**	Belgium	Walsin, Trou de la Naulette		Pre-LGM	No DNA		
**J4**	Belgium	Trou al'Wesse		Pleistocene	U. spelaeus		
**J5**	Belgium	Trou al'Wesse		Pleistocene	U. spelaeus		
**J6**	Belgium	Trou al'Wesse		Pleistocene	No DNA		
**J7**	Belgium	Trou al'Wesse		Pleistocene	No DNA		
**J9.1**	Belgium	Trou al'Wesse		Pleistocene	U. spelaeus		
**J9.2**	Belgium	Trou al'Wesse		Pleistocene	U. spelaeus		
**J10**	Belgium	Trou al'Wesse		Pleistocene	U. spelaeus		

**J11.2**	Belgium	Trou al'Wesse	Pleistocene	U. spelaeus	
**J14.1**	Belgium	Trou al'Wesse	Pleistocene	No DNA	
**J14.2**	Belgium	Trou al'Wesse	Pleistocene	U. spelaeus	
**J15**	Belgium	Trou al'Wesse	Pleistocene	U. spelaeus	
**J18**	UK (Wales)	Little Hoyle	LGM(?)	No DNA	
**J19**	UK (Wales)	Cae Gronw	ca. 35000	No DNA	
**J21**	UK (Wales)	Pontnewydd Cave	Pre-LGM	No DNA	
**J22**	UK (Wales)	Pontnewydd Cave	Pre-LGM	No DNA	
**J24**	UK (Wales)	Pontnewydd Cave	Pre-LGM	No DNA	
**J25**	UK (Wales)	Pontnewydd Cave	Pre-LGM	No DNA	
**J28**	UK (Wales)	Pontnewydd Cave	Pre-LGM	No DNA	
**J31**	UK (Wales)	Pontnewydd Cave	Pre-LGM	No DNA	
**J37**	UK (Wales)	Priory Farm Cave	Late Glacial	No DNA	
**J38**	UK (Wales)	Priory Farm Cave	Late Glacial	No DNA	
**R1**	Netherlands	Utrecht, Laagraven	LP-Early Holocene	No DNA	
**R2**	Netherlands	Gelderland, Wilp	Pleistocene-Weichselien	No DNA	
**R3**	Netherlands	Noord-Brabant, Empel, Maas	Late-Pleistocene	No DNA	
**R4**	Netherlands	North Sea, Maasvlakte 2	Late Pleistocene	No DNA	
**N1**	Netherlands	North Sea	LP-Early Holocene	No DNA	
**NS4**	Netherlands	North Sea	LP-Early Holocene	No DNA	
**NS7**	Netherlands	North Sea	LP-Early Holocene	No DNA	
**NS8**	Netherlands	North Sea	LP-Early Holocene	No DNA	
**NS10**	Netherlands	North Sea	LP-Early Holocene	No DNA	
**NS11**	Netherlands	North Sea	LP-Early Holocene	No DNA	
**NS12**	Netherlands	North Sea	LP-Early Holocene	No DNA	
**NS14**	Netherlands	North Sea	LP-Early Holocene	Partial	1b
**NS15**	Netherlands	North Sea	LP-Early Holocene	No DNA	
**NS16**	Netherlands	North Sea	LP-Early Holocene	No DNA	
**NS18**	Netherlands	North Sea	LP-Early Holocene	No DNA	

**NS21**	Netherlands	North Sea		LP-Early Holocene	No DNA
**NS22**	Netherlands	North Sea		LP-Early Holocene	No DNA
**NS24**	Netherlands	North Sea		LP-Early Holocene	No DNA
**NS25**	Netherlands	North Sea		LP-Early Holocene	No DNA
**NS26**	Netherlands	North Sea		LP-Early Holocene	No DNA
**NS27**	Netherlands	North Sea		LP-Early Holocene	No DNA
**NS28**	Netherlands	North Sea		LP-Early Holocene	No DNA
**NS29**	Netherlands	North Sea		LP-Early Holocene	No DNA
**NS30**	Netherlands	North Sea		LP-Early Holocene	No DNA
**NS31**	Netherlands	Ijssel river		LP-Early Holocene	No DNA
**Mz2**	Germany	Eich/Gimbsheim		LP-Early Holocene	No DNA
**Mz3**	Germany	Gimbsheim		LP-Early Holocene	No DNA
**Mz5**	Germany	Gimbsheim		LP-Early Holocene	No DNA
**Pl5**	Poland	Niedźwiedzia Cave		ca. 60 Ka (UTh)	U. spelaeus
**Pl6**	Poland	Cave no. 4 on Birów Hill		MIS 3-2	U. spelaeus
**Pl8**	Poland	Stajnia Cave		MIS 3	No DNA
**Pl10**	Poland	Radochowska Cave		MIS 2-1	U. spelaeus
**Pl12**	Poland	Biały Kamień Cave		MIS 4-3	No DNA
**Pl15**	Poland	Biśnik Cave		MIS 5-4	No DNA
**Pl20*[5]**	Poland	Kraków Spadzista B site	24360 ± 160		No DNA
**Pl21**	Poland	Kraków Spadzista E site		25-19 k	No DNA
**Sw2**	Sweden	Motala		?	No DNA
**Sw3**	Sweden	Österlöv		?	No DNA
**Sw5**	Sweden	Småland		?	No DNA
**Sw6**	Sweden	Skåne, Ugglarp	9355 ± 130		No DNA
**Sw7**	Sweden	Skåne, Breasten		ca 4300	No DNA
**Sw14**	Sweden	Skåne		?	No DNA
**Sw15**	Sweden	Skåne, Börringe		?	No DNA
**Sw16**	Sweden	Sörmland, Korsnäs		ca 4145	No DNA

**Sw17**	Sweden	Uppland, Åloppe	?	No DNA
**Sw18**	Sweden	Uppland, Åloppe	?	No DNA
**Sw20**	Sweden	Ögötland, Alvastra	?	No DNA
**Sw21**	Sweden	Stävie mosse	?	No DNA
**Sw22**	Sweden	Södra Skåne	?	No DNA
**Sw23**	Sweden	Skåne	?	No DNA
**Sw24**	Sweden	Skåne, Ingelstorp	?	No DNA
**Sw25**	Sweden	Skåne, Nöbbelöv	?	No DNA
**Sw26**	Sweden	Lund	?	No DNA
**Sw27**	Sweden	Skåne, Järavallen	?	No DNA
**Sw29**	Sweden	Skåne	?	No DNA
**Sw31**	Sweden	Skåne	?	No DNA
**Sw33**	Sweden	Skåne, Östra Vemmenhög	?	No DNA
**Sw34**	Sweden	Skåne, Gualöv	?	No DNA

References:

*[1a] Villaluenga, A. (2011). Sima de Illobi (Aralar, Navarra) yacimiento de oso pardo (U. arctos Linnaeus, 1758). Estudio taxonómico y tafonómico, Munibe Antropología-Arkeologia, 62: 145-174

[1b] Villaluenga, A. (2015). Restos esqueléticos de osos (Ursus arctos y Ursus spelaeus) en el oriente de la Región Cantábrica. Distribución geográfica y análisis biométrico. Munibe Antropologia-Arkeologia, 66(unknown), 77-92.

[1c] Villaluenga , A. (2016) Úrsidos en medios kársticos de la Cornisa Cantábrica (País Vasco y Navarra), *Estudio tafonómico de conjuntos arqueológicos y paleontológicos del Pleistoceno Superior y Holoceno*. BAR publishing (356p.).

*[2] Döppes, D., & Pacher, M. (2014). 10,000 years of Ursus arctos in the Alps–A success story? Analyses of the Late Glacial and Early Holocene brown bear remains from Alpine caves in Austria. *Quaternary International*, 339, 266-274.

*[3] Döppes, D., Rosendahl, W., Pacher, M., Imhof, W., Dalmeri, G., & Bocherens, H. (2008). Stabile Isotopenuntersuchungen an spätglazialen und holozänen

Braunbärenfunden aus Höhlen im Alpenraum. *Stalactite*, *58*(2), 64-66.

*[4] Nadachowski A., Żarski M., Urbanowski M., Wojtal P., Miękina B., Lipecki G., Ochman K., Krawczyk M., Jakubowski G., Tomek T. (2009). Late Pleistocene environment of the Częstochowa Upland (Poland) reconstructed on the basis of faunistic evidence from archaeological cave sites. *Institute of Systematics and Evolution of Animals, Polish Academy of Sciences*, Kraków, 112 pp.

*[5] Wojtal P., Wilczyński J., Nadachowski A., Münzel S. C. 2015. Gravettian hunting and exploitation of bears in Central Europe. *Quaternary International*, 359-360: 58-71.

Table S2.

Genbank ID	Country	Location	Age (^{14}C BP)	n	Age (cal. BP)	Age (Year)	Haplotype	(Sub-) Clade	$\delta^{13}C$	$\delta^{15}N$	Alt. (m)	Publication
**AJ809333**	Austria	Ramesch	47420±1000	1	51184 ± 2166		3.23	3				Hofreiter et al. 2004
**AJ809334**	Austria	Winden	40020±1000	1	43833±874		1b1	1b				Hofreiter et al. 2004
**EF488487**	Spain	Cuevas del Somo	7500±55	1	8304±67		1a21	1a				Valdiosera et al. 2007
**EF488488**	Italy	Abruzzo	16440±65	1	19747±293		1a20	1a				Valdiosera et al. 2007
**EF488490**	Spain	Cuevas del Somo	5380±45	1	6167±91		1a21	1a				Valdiosera et al. 2007
**EF488491**	France	Mt. Ventoux	1790±55	1	1718±79		1a26	1a	-19.3	3.7	1650	Valdiosera et al. 2007
**EF488492**	France	Mt. Ventoux	3845±40	1	4276±85		1c3	1c	-19.7	3.7	1650	Valdiosera et al. 2007
**EF488493**	France	Mt. Ventoux	1750±30	1	1662±40		1b28	1b	-19.6	4.1	1650	Valdiosera et al. 2007
**EF488494**	France	Mt. Ventoux	6525±50	1	7434±50		1c2	1c				Valdiosera et al. 2007
**EF488495**	France	Mt. Ventoux	1570±35	1	1467±44		1a26	1a				Valdiosera et al. 2007
**EF488496**	France	Mt. Ventoux	4645±40	1	5391±57		1c4	1c				Valdiosera et al. 2007
**EF488497**	Spain	Navacepada	350±40	1	405±63		1a22	1a				Valdiosera et al. 2007
**EF488498**	Germany	Mühlberg	1770±35	1	1690±54		3.1	3				Valdiosera et al. 2007
**EF488499**	Germany	Bad Frankenhausen	5210±35	1	5965±28		1b25	1b				Valdiosera et al. 2007
**EF488500**	Germany	Wysburg	750±150	1	728±135		1b25	1b				Valdiosera et al. 2007
**EF488501**	Germany	Dienstedt	1665±35	1	1580±38		1b32	1b				Valdiosera et al. 2007
**EF488503**	Spain	Cuevas del Somo	4624±45	1	5381±62		1a21	1a				Valdiosera et al. 2007
**EF488504**	Spain	Atapuerca	17440±425	1	20881±318		1e1	1e				Valdiosera et al. 2007
**EF488505**	France	Mt. Ventoux	3445±40	1	3730±72		1b25	1b				Valdiosera et al. 2007
**EF488506**	Romania	Pestera Baltagul	2250±50	1	2253±69		3.9	3				Valdiosera et al. 2007
**EF488507**	Romania	Pestera Baltagul	2250±50	1	2253±69		3.9	3				Valdiosera et al. 2007
**EU400179**	Spain	Burgos	6325±50	1	7254±57		1a21	1a				Valdiosera et al. 2008
**EU400181**	Spain	Valdegoba		1	80 k*		1e1	1e				Valdiosera et al. 2008
**EU400182**	Spain	Valdegoba		1	80 k*		1e3	1e				Valdiosera et al. 2008

**EU400183**	Spain	Valdegoba	75-90 ka	1	80 k*		1e2	1e				Valdiosera et al. 2008
**EU400207**	Spain	Asturias		1		1910 AD	1a12	1a				Valdiosera et al. 2008
**EU400180**	Spain	Arlanpe	40-80 ka	1	60 k*		3.26	3				Valdiosera et al. 2008
**FN292971**	Lebanon			1		1870 AD	1d1	1d				Calvignac et al. 2009
**JF900158**	England	Carsington	12143±46	1	13936±146		1a7	1a	-18.9	5.8	300	Edwards et al. 2011
**JF900159**	Ireland	Limerick	10650±100	1	12569±174		2.6	2	-21.2	7.0	N/A	Edwards et al. 2011
**JF900160**	Ireland	Kesh Corran	11920±85	1	13683±182		Partial	2	-17.8	6.7	N/A	Edwards et al. 2011
**JF900161**	Ireland	Cork	26340±32	1	30937±254		Partial	2	-16.1	9.6	N/A	Edwards et al. 2011
**JF900162**	Ireland	Cork	26340±32	1	30937±254		2.7	2	-21.5	9.5	N/A	Edwards et al. 2011
**JF900163**	Ireland	Cork	33310±770	1	39055±1384		1b9	1b	-19.7	5.9	100	Edwards et al. 2011
**JF900164**	Ireland	Cork	37870±1270	1	42618±628		1b2	1b	-20.2	6.0	100	Edwards et al. 2011
**JF900165**	Ireland	Waterford	32430±670	1	37818±915		2.1	2	-18.7	7.2	N/A	Edwards et al. 2011
**JF900166**	Ireland	Leitrim	4520±37	1	5180±93		1a17	1a	-21.6	8.8	N/A	Edwards et al. 2011
**JF900167**	Ireland	Leitrim	3517±31	1	3791±50		1a30	1a	N/A	N/A		Edwards et al. 2011
**JF900168**	Ireland	Kesh Corran	11460±57	1	13219±105		2.4	2	-17.9	6.5	N/A	Edwards et al. 2011
**JF900169**	Ireland	Limerick	8719±48	1	9702±96		2.3	2	-21.4	6.8	N/A	Edwards et al. 2011
**JF900170**	Ireland	Clare	10495±51	1	12393±199		2.5	2	-19.9	6.1	N/A	Edwards et al. 2011
**JF900171**	Ireland	Cork	32648±245	1	37970±613		Partial	1b	-20.0	5.5	N/A	Edwards et al. 2011
**JF900172**	Ireland	Clare	9946±53	1	11391±125		2.2	2	-18.5	9.4	N/A	Edwards et al. 2011
**JF900173**	Ireland	Waterford	28390±177	1	33067±625		2.1	2	-19.1	7.6	N/A	Edwards et al. 2011
**JF900174**	Ireland	Sligo	4136±37	1	4687±94		1a16	1a	-21.1	4.6	N/A	Edwards et al. 2011
**JF900175**	Ireland	Leitrim	2956±33	1	3126±60		1a29	1a	-21.7	4.6	N/A	Edwards et al. 2011
	England	Star Carr	9533±37	1	10890±190		Partial	1a	-20.8	5.8	<100	Edwards et al. 2014
	England	Kinsey Cave	12535±55	1	14645 ±367		1a7	1a	N/A	N/A		Edwards et al. 2014
	England	Kinsey Cave	12465±65	1	14554 ±366		Partial	1a	-18.8	5.2	N/A	Edwards et al. 2014
	England	Victoria Cave	10080±55	1	11613±222		Partial	1a	-18.9	5.2	440	Edwards et al. 2014
	England	Victoria Cave	9594±40	1	10945±189		Partial	1a	-19.0	4.7	440	Edwards et al. 2014
	England	Victoria Cave	12520±50	1	14626±358		1a7	1a	-18.9	7.6	440	Edwards et al. 2014
	England	Victoria Cave	12250±55	1	14158±216		1a1	1a	-19.0	5.9	440	Edwards et al. 2014
	England	Victoria Cave	12125±50	1	13971±73		1a1	1a	N/A	N/A		Edwards et al. 2014

	England	Victoria Cave	12490±50	1	14586±352		1a7	1a	N/A	N/A		Edwards et al. 2014
	England	Raven Scar Cave	9000±40	1	10188±54		1a1	1a	-21.6	0.8	320	Edwards et al. 2014
	England	Gr. Kelco Cave	10355±55	1	12194±202		Partial	No DNA	-20.4	7.7	200	Edwards et al. 2014
	England	Sewell's Cave	11690±45	1	13546±135		1a7	1a	-19.0	5.2	N/A	Edwards et al. 2014
	England	Sewell's Cave	12230±50	1	14089±149		1a1	1a	-18.5	6.1	N/A	Edwards et al. 2014
	England	Conistone Dib Cave	11655±45	1	13515±137		Partial	1a	-19.3	6.1	N/A	Edwards et al. 2014
**AY082845/81**	Scotland	Bear Cave	2673±54	1	2801±80		1a5	1a	N/A	N/A		Barnes et al. 2002
**JQ823238**	Denmark		5310±20	1	6098±62		1b25	1b				Bray et al. 2013
**JQ823240**	Norway		3150±120	1	3359±149		3.1	3				Bray et al. 2013
**JQ823242**	Norway		6210±100	1	7109±123		3.5	3				Bray et al. 2013
**JQ823243**	Norway		2800±160	1	2974±190		3.1	3				Bray et al. 2013
**JQ823244**	Norway		2870±80	1	3022±117		3.1	3				Bray et al. 2013
**JQ823250**	Norway		1310±30	1	1243±40		3.7	3				Bray et al. 2013
**JQ823245**	Norway		3530±110	1	3827±141		3.1	3				Bray et al. 2013
**JQ823252**	Norway		4420±70	1	5072±148		3.1	3				Bray et al. 2013
**JQ823256**	Norway		3980±180	1	4451±263		3.1	3				Bray et al. 2013
**JQ823257**	Norway		3970±60	1	4422±91		3.1	3				Bray et al. 2013
	Norway			10	Holocene		3.1	3				Bray et al. 2013
**FR733658**	Germany	Geissenklösterle	30320±190	1	34876±276		Partial	3	-19.3	9.1	580	Münzel et al. 2011
**FR733658**	Germany	Geissenklösterle	30560±180	1	35008±358		Partial	3	-19.5	8.3	580	Münzel et al. 2011
**FR733658**	Germany	Hohle Fels	14600±60	1	17743±286		Partial	3			450	Münzel et al. 2011
	Sweden	Korsnäs	4145±40	1	4699±90			No DNA	-20.0	3.8	<100	Fornander et al. 2008
	Sweden	Unna Saiva	1398±56	1	1323±36			No DNA	-20.6	6.4	490	Salmi et al. 2015
**KM886400**	Sweden	Arjeplog		1		1939 AD	3.1	3				Xenikoudakis et al. 2015
**KM886401**	Sweden	Jokkmokk		1		1939 AD	3.1	3				Xenikoudakis et al. 2015
**KM886402**	Sweden	Porjus		1		1939 AD	3.1	3				Xenikoudakis et al. 2015
**KM886403**	Sweden	Dorotea		1		1940 AD	3.2	3				Xenikoudakis et al. 2015
**KM886404**	Sweden	Murjek		1		1940 AD	3.1	3				Xenikoudakis et al. 2015
**KM886405**	Sweden	Kåitokvagga		1		1940 AD	3.2	3				Xenikoudakis et al. 2015

**KM886406**	Sweden	Dorotea	1	1940 AD	3.2	3	Xenikoudakis et al. 2015
**KM886407**	Sweden	Norråker	1	1940 AD	3.2	3	Xenikoudakis et al. 2015
**KM886408**	Sweden	Sonfjället	1	1928 AD	1a13	1a	Xenikoudakis et al. 2015
**KM886409**	Sweden	Paittasjärvi	1	1928 AD	3.1	3	Xenikoudakis et al. 2015
**KM886410**	Sweden	Gällivare	1	1932 AD	3.1	3	Xenikoudakis et al. 2015
**KM886411**	Sweden	Kiruna	1	1910 AD	3.1	3	Xenikoudakis et al. 2015
**KM886412**	Norway	Hatfjelddalen	1	1915 AD	3.2	3	Xenikoudakis et al. 2015
**KM886413**	Norway	Southern part	1	1905 AD	1a2	1a	Xenikoudakis et al. 2015
**KM886414**	Norway	Telemark fylke	1	1907 AD	1a15	1a	Xenikoudakis et al. 2015
**KM886415**	Norway	Numedal	1	1905 AD	1a6	1a	Xenikoudakis et al. 2015
**KM886416**	Norway	Rendalen	1	1859 AD	1a13	1a	Xenikoudakis et al. 2015
**KM886417**	Sweden	Dalarna	1	1864 AD	1a13	1a	Xenikoudakis et al. 2015
**KM886418**	Sweden	Värmland	1	1832 AD	1a2	1a	Xenikoudakis et al. 2015
**KM886419**	Sweden	Avaträsk	1	1915 AD	3.7	3	Xenikoudakis et al. 2015
**KM886420**	Sweden	Jämtland	1	1832 AD	3.1	3	Xenikoudakis et al. 2015
**KM886421**	Sweden	Dalarna	1	1864 AD	1a3	1a	Xenikoudakis et al. 2015
**KM886422**	Sweden	Jämtland	1	1846 AD	1a13	1a	Xenikoudakis et al. 2015
**KM886423**	Sweden	Kaalasluspa	1	1909 AD	3.1	3	Xenikoudakis et al. 2015
**KM886424**	Sweden	Dalarna	1	1864 AD	1a13	1a	Xenikoudakis et al. 2015
**KM886425**	Sweden	Jämtland	1	1842 AD	1a13	1a	Xenikoudakis et al. 2015
**KM886426**	Sweden	Vuoggatjålme	1	1909 AD	3.1	3	Xenikoudakis et al. 2015
**KM886427**	Sweden	Dalarna	1	1864 AD	1a13	1a	Xenikoudakis et al. 2015
**KM886428**	Sweden	Dalarna	1	1864 AD	1a3	1a	Xenikoudakis et al. 2015
**KM886429**	Sweden	Nordansjö	1	1830 AD	1a1	1a	Xenikoudakis et al. 2015
**KM886430**	Sweden	Värmland	1	1832 AD	1a1	1a	Xenikoudakis et al. 2015
**KM886431**	Norway	Ulvdalen	1	1899 AD	1a2	1a	Xenikoudakis et al. 2015
**KM886432**	Norway	Hjartdal	1	1905 AD	1a6	1a	Xenikoudakis et al. 2015
**KM886433**	Sweden	Dalarna	1	1835 AD	1.1	3	Xenikoudakis et al. 2015
**KM886434**	Sweden	Vilhelmina	1	1939 AD	3.2	3	Xenikoudakis et al. 2015
**KM886435**	Sweden	Jämtland	1	1922 AD	3.2	3	Xenikoudakis et al. 2015

**KM886436**	Sweden	Gällivare		1	1940 AD	3.1	3	Xenikoudakis et al. 2015
**KM886437**	Sweden	Värmland		1	1835 AD	1a2	1a	Xenikoudakis et al. 2015
**KM886438**	Sweden	Dalarna		1	1869 AD	1a8	1a	Xenikoudakis et al. 2015
**KM886439**	Norway	Hjartdal		1	1905 AD	1a6	1a	Xenikoudakis et al. 2015
**KM886440**	Norway	Etnedal		1	1899 AD	1a2	1a	Xenikoudakis et al. 2015
**KM886441**	Sweden	Norrland		1	1865 AD	1a11	1a	Xenikoudakis et al. 2015
**KM886442**	Sweden	Värmland		1	1844 AD	1a5	1a	Xenikoudakis et al. 2015
**KM886443**	Sweden	Bohuslän		1	1845 AD	1a1	1a	Xenikoudakis et al. 2015
**KM886444**	Sweden	Värmland		1	1847 AD	1a5	1a	Xenikoudakis et al. 2015
**KM886445**	Sweden	Dalarna		1	1848 AD	1a1	1a	Xenikoudakis et al. 2015
**KM886446**	Sweden	Värmland		1	1880 AD	1a2	1a	Xenikoudakis et al. 2015
**KM886448**	Sweden	Värmland		1	1927 AD	1a2	1a	Xenikoudakis et al. 2015
**KM886449**	Sweden	Värmland		1	1927 AD	1a8	1a	Xenikoudakis et al. 2015
**KM886450**	Sweden	Västergötland		1	1838 AD	1a1	1a	Xenikoudakis et al. 2015
**KM886451**	Norway	Bergen		1	1895 AD	1a2	1a	Xenikoudakis et al. 2015
**KM886452**	Sweden	Närke		1	1860 AD	1a5	1a	Xenikoudakis et al. 2015
**KM886453**	Sweden	Dalsland		1	1840 AD	1a1	1a	Xenikoudakis et al. 2015
**KM886454**	Sweden	Värmland		1	1845 AD	1a1	1a	Xenikoudakis et al. 2015
**KM886455**	Sweden	Värmland		1	1800 AD	1a2	1a	Xenikoudakis et al. 2015
**KM886456**	Sweden	Värmland		1	1869 AD	1a8	1a	Xenikoudakis et al. 2015
**KM886457**	Norway	Unknown		1	1862 AD	1a6	1a	Xenikoudakis et al. 2015
**KM886458**	Sweden	Nedre Vaptsjön		1	Medieval	3.1	3	Xenikoudakis et al. 2015
**KM886459**	Sweden	Unna Saiva		1	Medieval	3.1	3	Xenikoudakis et al. 2015
**X75862**	Italy	Abruzzo	modern	4		1b25	1b	Taberlet & Bouvet 1994
**X75864**	Bulgaria		modern	1		1b32	1b	Taberlet & Bouvet 1994
**X75865**	Spain	Cantabria	modern	1		1a12	1a	Taberlet & Bouvet 1994
**X75866**	Spain	Cantabria	modern	1		1a12	1a	Taberlet & Bouvet 1994
**X75867**	Croatia		modern	1		1b26	1b	Taberlet & Bouvet 1994
**X75868**	Sweden	Dalarna	modern	7		1a13	1a	Taberlet & Bouvet 1994
**X75869**	Estonia		modern	2		3.12	3	Taberlet & Bouvet 1994

**X75870**	Greece		modern	1	1b31	1b	Taberlet & Bouvet 1994
**X75871**	Norway		modern	1	1a1	1a	Taberlet & Bouvet 1994
**X75872**	Romania		modern	1	3.19	3	Taberlet & Bouvet 1994
**X75873**	Romania		modern	3	3.9	3	Taberlet & Bouvet 1994
**X75874**	Russia		modern	1	3.1	3	Taberlet & Bouvet 1994
**X75874**	Estonia		modern	2	3.1	3	Taberlet & Bouvet 1994
**X75874**	Finland		modern	2	3.1	3	Taberlet & Bouvet 1994
**X75874**	Sweden	Norrland	modern	7	3.1	3	Taberlet & Bouvet 1994
**X75875**	Slovakia		modern	1	3.1	3	Taberlet & Bouvet 1994
**X75876**	Slovakia		modern	1	3.1	3	Taberlet & Bouvet 1994
**X75877**	Slovenia		modern	13	1b25	1b	Taberlet & Bouvet 1994
**X75877**	Croatia		modern	1	1b25	1b	Taberlet & Bouvet 1994
**X75877**	Bosnia		modern	2	1b25	1b	Taberlet & Bouvet 1994
**X75877**	Italy	Trentino	modern	1	1b25	1b	Taberlet & Bouvet 1994
**X75878**	France	Pyrenées	modern	4	1a26	1a	Taberlet & Bouvet 1994
**EU497665**	France	Pyrenées	modern	1	1a28	1a	Bon et al. 2008
	Romania		modern	1	1b34	1b	Zachos et al. 2008
	Romania		modern	1	3.1	3	Zachos et al. 2008
	Romania		modern	4	1b32	1b	Zachos et al. 2008
	Romania		modern	1	3.7	3	Zachos et al. 2008
	Romania		modern	1	1b34	1b	Zachos et al. 2008
	Romania		modern	1	1b32	1b	Zachos et al. 2008
	Romania		modern	1	1b32	1b	Zachos et al. 2008
	Romania		modern	1	1b33	1b	Zachos et al. 2008
	Romania		modern	2	3.9	3	Zachos et al. 2008
	Romania		modern	3	1b32	1b	Zachos et al. 2008
	Italy		modern	17	1b25	1b	Zachos et al. 2008
**EU400184-207**	Spain	Cantabria	modern	21	1a12	1a	Valdiosera et al. 2008
**EU400197**	Spain	Cantabria	modern	1	1a12	1a	Valdiosera et al. 2008

**EU400202**	Spain	Cantabria	modern	1		1a19	1a		Valdiosera et al. 2008
**EU400205**	Spain	Cantabria	modern	1		1a18	1a		Valdiosera et al. 2008
**HQ602651**	Croatia		modern	45		1b25	1b		Kocijan et al. 2011
**HQ602652**	Croatia		modern	13		1b29	1b		Kocijan et al. 2011
**HQ602653**	Croatia		modern	1		1b26	1b		Kocijan et al. 2011
**AP012588**	Russia	Urals	modern	1		3.1	3		Hirata et al. 2013
**AP012590**	Bulgaria		modern	1		3.9	3		Hirata et al. 2013
**AP012591**	Bulgaria		modern	1		1b35	1b		Hirata et al. 2013
**KJ638591**	Bulgaria		modern	5		1b34	1b		Frosch et al. 2014
**KJ638592**	Bulgaria		modern	53		1b35	1b		Frosch et al. 2014
**KJ638593**	Bulgaria		modern	10		1b36	1b		Frosch et al. 2014
**KJ638594**	Bulgaria		modern	8		1a33	1a		Frosch et al. 2014
**KJ638595**	Bulgaria		modern	4		3.8	3		Frosch et al. 2014
**KJ638596**	Bulgaria		modern	1		3.10	3		Frosch et al. 2014
**KJ638597**	Bulgaria		modern	2		3.20	3		Frosch et al. 2014
	Bulgaria		modern	9		1b32	1b		Frosch et al. 2014
	Bulgaria		modern	8		3.9	3		Frosch et al. 2014
	Bulgaria		modern	1		3.1	3		Frosch et al. 2014
	Bulgaria		modern	1		3.7	3		Frosch et al. 2014
	Turkey	Akseki	modern	1		1d2	1d		Cilingir et al. 2015
	Turkey	Akseki	modern	1		1b30	1b		Cilingir et al. 2015
	Turkey	Bursa	modern	1		1b29	1b		Cilingir et al. 2015
**EU526765**	Estonia		modern	1		3.1	3		Korsten et al. 2009
**EU526765**	Finland		modern	6		3.1	3		Korsten et al. 2009
**EU526765**	Russia		modern	52		3.1	3		Korsten et al. 2009
**EU526766**	Russia		modern	1		3.1	3		Korsten et al. 2009
**EU526767**	Finland		modern	2		3.14	3		Korsten et al. 2009
**EU526768**	Russia		modern	1		3.1	3		Korsten et al. 2009
**EU526769**	Russia		modern	1		3.7	3		Korsten et al. 2009
**EU526770**	Finland		modern	4		3.1	3		Korsten et al. 2009

**EU526770**	Russia	modern	4	3.1	3	Korsten et al. 2009
**EU526771**	Russia	modern	2	3.1	3	Korsten et al. 2009
**EU526772**	Russia	modern	7	3.1	3	Korsten et al. 2009
**EU526773**	Finland	modern	1	3.1	3	Korsten et al. 2009
**EU526774**	Russia	modern	1	3.1	3	Korsten et al. 2009
**EU526776**	Russia	modern	2	3.1	3	Korsten et al. 2009
**EU526777**	Finland	modern	7	3.1	3	Korsten et al. 2009
**EU526777**	Russia	modern	1	3.1	3	Korsten et al. 2009
**EU526778**	Finland	modern	1	3.3	3	Korsten et al. 2009
**EU526779**	Finland	modern	1	3.1	3	Korsten et al. 2009
**EU526780**	Finland	modern	1	3.1	3	Korsten et al. 2009
**EU526781**	Russia	modern	3	3.1	3	Korsten et al. 2009
**EU526782**	Russia	modern	1	3.6	3	Korsten et al. 2009
**EU526783**	Russia	modern	3	3.1	3	Korsten et al. 2009
**EU526784**	Estonia	modern	27	3.1	3	Korsten et al. 2009
**EU526785**	Estonia	modern	4	3.1	3	Korsten et al. 2009
**EU526785**	Russia	modern	2	3.1	3	Korsten et al. 2009
**EU526786**	Russia	modern	1	3.1	3	Korsten et al. 2009
**EU526787**	Russia	modern	1	3.1	3	Korsten et al. 2009
**EU526788**	Russia	modern	3	3.1	3	Korsten et al. 2009
**EU526789**	Russia	modern	1	3.1	3	Korsten et al. 2009
**EU526791**	Russia	modern	3	3.1	3	Korsten et al. 2009
**EU526792**	Finland	modern	3	3.1	3	Korsten et al. 2009
**EU526793**	Finland	modern	1	3.1	3	Korsten et al. 2009
**EU526793**	Russia	modern	2	3.1	3	Korsten et al. 2009
**EU526799**	Finland	modern	1	3.11	3	Korsten et al. 2009
**EU526801**	Estonia	modern	2	3.1	3	Korsten et al. 2009
**EU526802**	Estonia	modern	1	3.12	3	Korsten et al. 2009
**HE657199**	Romania	modern	1	1b27	1b	Hailer et al. 2012
**HE657200**	Romania	modern	1	1b34	1b	Hailer et al. 2012

**HE657201**	Bulgaria	modern	1	3.1	3	Hailer et al. 2012
**HE657203**	Romania	modern	1	3.1	3	Hailer et al. 2012
**HE657208**	Norway	modern	1	3.22	3	Hailer et al. 2012
**HE657209**	Norway	modern	1	3.22	3	Hailer et al. 2012
**HE657210**	Romania	modern	1	3.7	3	Hailer et al. 2012
**HE657211**	Romania	modern	1	3.9	3	Hailer et al. 2012
**HE657212**	Sweden	modern	1	3.2	3	Hailer et al. 2012
**HE657213**	Romania	modern	1	1b34	1b	Hailer et al. 2012
**HE657214**	Romania	modern	1	1b32	1b	Hailer et al. 2012
**HE657215**	Norway	modern	1	3.22	3	Hailer et al. 2012
**HE657216**	Norway	modern	1	3.22	3	Hailer et al. 2012
	Sweden	modern	13	3.1	3	Xenikoudakis et al. 2015
	Sweden	modern	14	3.2	3	Xenikoudakis et al. 2015
	Sweden	modern	20	1a13	1a	Xenikoudakis et al. 2015

* Estimated age

References:

Bon, C., Caudy, N., de Dieuleveult, M., Fosse, P., Philippe, M., Maksud, F., ... & Elalouf, J-M. (2008). Deciphering the complete mitochondrial genome and phylogeny of the extinct cave bear in the Paleolithic painted cave of Chauvet. *Proceedings of the National Academy of Sciences*, *105*(45), 17447-17452.

Barnes, I., Matheus, P., Shapiro, B., Jensen, D., & Cooper, A. (2002). Dynamics of Pleistocene population extinctions in Beringian brown bears. *Science*, *295*(5563), 2267-2270.

Bray, S. C., Austin, J. J., Metcalf, J. L., Østbye, K., Østbye, E., Lauritzen, S. E., ... & Cooper, A. (2013). Ancient DNA identifies post-glacial recolonisation, not recent bottlenecks, as the primary driver of contemporary mt DNA phylogeography and diversity in Scandinavian brown bears. *Diversity and Distributions*, *19*(3), 245-256.

Calvignac, S., Hughes, S., & Hänni, C. (2009). Genetic diversity of endangered brown bear (Ursus arctos) populations at the crossroads of Europe, Asia and Africa. *Diversity and Distributions*, *15*(5), 742-750.

Çilingir, F. G., Akın Pekşen, Ç., Ambarlı, H., Beerli, P., & Bilgin, C. C. (2016). Exceptional maternal lineage diversity in brown bears (Ursus arctos) from Turkey. *Zoological Journal of the Linnean Society*, *176*(2), 463-477.

Edwards, C. J., Suchard, M. A., Lemey, P., Welch, J. J., Barnes, I., Fulton, T. L., ... & Valdiosera, C. E. (2011). Ancient hybridization and an Irish origin for the modern polar bear matriline. *Current Biology*, *21*(15), 1251-1258.

Edwards, C. J., Ho, S. Y., Barnett, R., Coxon, P., Bradley, D. G., Lord, T. C., & O'Connor, T. (2014). Continuity of brown bear maternal lineages in northern England through the Last-glacial period. *Quaternary Science Reviews*, *96*, 131-139.

Fornander, E., Eriksson, G., & Lidén, K. (2008). Wild at heart: Approaching Pitted Ware identity, economy and cosmology through stable isotopes in skeletal material from the Neolithic site Korsnäs in Eastern Central Sweden. *Journal of Anthropological Archaeology*, *27*(3), 281-297.

Frosch, C., Dutsov, A., Zlatanova, D., Valchev, K., Reiners, T. E., Steyer, K., ... & Nowak, C. (2014). Noninvasive genetic assessment of brown bear population structure in Bulgarian mountain regions. *Mammalian Biology-Zeitschrift für Säugetierkunde*, *79*(4), 268-276.

Hailer, F., Kutschera, V. E., Hallström, B. M., Klassert, D., Fain, S. R., Leonard, J. A., ... & Janke, A. (2012). Nuclear genomic sequences reveal that polar bears are an old and distinct bear lineage. *Science*, *336*(6079), 344-347.

Hirata, D., Mano, T., Abramov, A. V., Baryshnikov, G. F., Kosintsev, P. A., Vorobiev, A. A., ... & Fukui, D. (2013). Molecular phylogeography of the brown bear (Ursus arctos) in northeastern Asia based on analyses of complete mitochondrial DNA sequences. *Molecular biology and evolution*, *30*(7), 1644-1652.

Hofreiter, M., Serre, D., Rohland, N., Rabeder, G., Nagel, D., Conard, N., ... & Pääbo, S. (2004). Lack of phylogeography in European mammals before the last glaciation. *Proceedings of the National Academy of Sciences*, *101*(35), 12963-12968.

Kocijan, I., Galov, A., Ćetković, H., Kusak, J., Gomerčić, T., & Huber, Đ. (2011). Genetic diversity of Dinaric brown bears (Ursus arctos) in Croatia with implications for bear conservation in Europe. *Mammalian Biology-Zeitschrift für Säugetierkunde*, *76*(5), 615-621.

Korsten, M., Ho, S. Y., Davison, J., PÄHN, B., Vulla, E., Roht, M., ... & Pilot, M. (2009). Sudden expansion of a single brown bear maternal lineage across northern continental Eurasia after the last ice age: a general demographic model for mammals?. *Molecular Ecology*, *18*(9), 1963-1979.

Münzel, S. C., Stiller, M., Hofreiter, M., Mittnik, A., Conard, N. J., & Bocherens, H. (2011). Pleistocene bears in the Swabian Jura (Germany): genetic replacement, ecological displacement, extinctions and survival. *Quaternary International*, *245*(2), 225-237.

Salmi, A. K., Äikäs, T., Fjellström, M., & Spangen, M. (2015). Animal offerings at the Sámi offering site of Unna Saiva–changing religious practices and human–animal relationships. *Journal of Anthropological Archaeology*, *40*, 10-22.

Taberlet, P., & Bouvet, J. (1994). Mitochondrial DNA polymorphism, phylogeography, and conservation genetics of the brown bear Ursus arctos in Europe. *Proc. R. Soc. Lond. B*, *255*(1344), 195-200.

Valdiosera, C. E., García, N., Anderung, C., Dalén, L., Crégut-Bonnoure, E., Kahlke, R. D., ... & Götherström, A. (2007). Staying out in the cold: glacial refugia and mitochondrial DNA phylogeography in ancient European brown bears. *Molecular ecology*, *16*(24), 5140-5148.

Valdiosera, C. E., García-Garitagoitia, J. L., Garcia, N., Doadrio, I., Thomas, M. G., Hänni, C., ... & Götherström, A. (2008). Surprising migration and population size dynamics in ancient Iberian brown bears (Ursus arctos). *Proceedings of the National Academy of Sciences*, *105*(13), 5123-5128.

Xenikoudakis, G., Ersmark, E., Tison, J. L., Waits, L., Kindberg, J., Swenson, J. E., & Dalén, L. (2015). Consequences of a demographic bottleneck on genetic structure and variation in the Scandinavian brown bear. *Molecular ecology*, *24*(13), 3441-3454.

Zachos, F. E., Otto, M., Unici, R., Lorenzini, R., & Hartl, G. B. (2008). Evidence of a phylogeographic break in the Romanian brown bear (Ursus arctos) population from the Carpathians. *Mammalian Biology-Zeitschrift für Säugetierkunde*, *73*(2), 93-101.
