# Supplementary material for: Genetic turnovers and northern survival during the last glacial maximum in European brown bears
Source: Ecol Evol. 2019 Apr 16;9(10):5891–905. doi: 10.1002/ece3.5172 (PMC6540696; doi:10.1002/ece3.5172)

Table S3.

1. Arct_1F (5'- 3') TAAACTATTCCCTGGTACA
   H16299 (5'- 3') GGAGCGAGAAGAGGTACACGT
   (ca 270 bp exl. primers)

2. Arct_1F (5'- 3') TAAACTATTCCCTGGTACA
   OP55R (5'- 3') CTCCCGGACTAAGTGAAATACATG
   (ca 209 bp exl. primers)

3. Arct_1F (5'- 3') TAAACTATTCCCTGGTACA
   Arct_1R (5'- 3') AGGTATTCGAGGACATAC
   (61 bp exl. primers)

4. L16164 (5'- 3') GCCCCATGCATATAAGCATG
   H16299 (5'- 3') GGAGCGAGAAGAGGTACACGT
   (132 bp exl. primers)

5. OP55F (5'- 3') CCCCATGCATATAAGCATGTACAT
   OP55R (5'- 3') CTCCCGGACTAAGTGAAATACATG
   (67 bp exl. primers)

6. OP54F (5'- 3') GTATAGTCTGTAACGATGTAT
   OP54R (5'- 3') AGGTACACGTACTCGCAA
   (55 bp exl. primers)

Figure below shows coverage of primer pairs: Black bar at bottom represents entire fragment of the mitochondrial control region, spanning ca. 270 bp. Dotted line indicates a ca. 70 bp stretch of repetitions and extremely slight variation, which was not included.

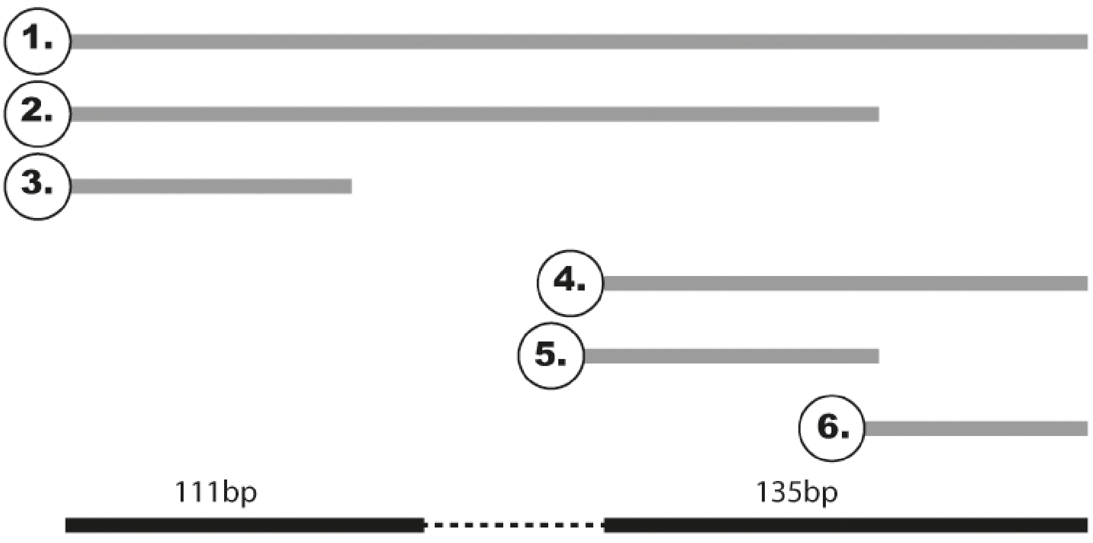

Supplement: Supplementary file 2 [file ECE3-9-5891-s002.pdf]
