# Supplementary material for: Genetic turnovers and northern survival during the last glacial maximum in European brown bears
Source: Ecol Evol. 2019 Apr 16;9(10):5891–905. doi: 10.1002/ece3.5172 (PMC6540696; doi:10.1002/ece3.5172)
Supplement: Supplementary file 3 [file ECE3-9-5891-s003.pdf]

ID	Country	GenBank	Location	Age (^{14}C BP)	Age (cal. BP)	Age (indirect)	Haplotype	(Sub-) Clade	δ13C	δ15N	Alt. (m)	n	Publication
**Mj7**	Belgium		Goyet, 3e caverne	19770 ± 130 (OxA-32251)	23675 ± 298		1a1	1a	-19.8	4.2	130	1	
**Pa19**	France		Igue du Gral	19390 ± 100	23155 ± 283		1a1	1a				1	
**J11.1**	Belgium		Trou al'Wesse	17170 ± 90 (OxA-32186)	20609 ± 303		1a1	1a	-19.3	4.1	200	1	
	Italy	EF488488	Abruzzo	16440±65	19747±293		1a20	1a				1	Valdiosera et al. 2007
	England		Kinsey Cave	12535±55	14645 ±367		1a7	1a	N/A	N/A		1	Edwards et al. 2014
	England		Victoria Cave	12520±50	14626±358		1a7	1a	-18.9	7.6	440	1	Edwards et al. 2014
**Aix5**	France		Doubs	12390 ± 30	14603 ± 327		1a14	1a				1	
	England		Victoria Cave	12490±50	14586±352		1a7	1a	N/A	N/A		1	Edwards et al. 2014
	England		Kinsey Cave	12465±65	14554 ±366		Partial	1a	-18.8	5.2	N/A	1	Edwards et al. 2014
	England		Victoria Cave	12250±55	14158±216		1a1	1a	-19.0	5.9	440	1	Edwards et al. 2014
	England		Sewell's Cave	12230±50	14089±149		1a1	1a	-18.5	6.1	N/A	1	Edwards et al. 2014
	England		Victoria Cave	12125±50	13971±73		1a1	1a	N/A	N/A		1	Edwards et al. 2014
	England	JF900158	Carsington	12143±46	13936±146		1a7	1a	-18.9	5.8	300	1	Edwards et al. 2011
	England		Sewell's Cave	11690±45	13546±135		1a7	1a	-19.0	5.2	N/A	1	Edwards et al. 2014
	England		Conistone Dib Cave	11655±45	13515±137		Partial	1a	-19.3	6.1	N/A	1	Edwards et al. 2014
**Ro9**	Romania		Southern Carpathians	10205 ± 55 (OxA-30295)	11903 ± 147		1a1	1a	-19.3	2.6	N/A	1	
	England		Victoria Cave	10080±55	11613±222		Partial	1a	-18.9	5.2	440	1	Edwards et al. 2014
	England		Victoria Cave	9594±40	10945±189		Partial	1a	-19.0	4.7	440	1	Edwards et al. 2014
	England		Star Carr	9533±37	10890±190		Partial	1a	-20.8	5.8	<100	1	Edwards et al. 2014
**Sw4**	Sweden		Smedstorp, Skåne	9105 ± 50 (OxA-32192)	10290 ± 57		1a1	1a	-20.7	4.5	<100	1	
	England		Raven Scar Cave	9000±40	10188±54		1a1	1a	-21.6	0.8	320	1	Edwards et al. 2014
**J34**	UK (Wales)		Penwyllt Cave			Late Glacial	1a7	1a				1	
**J35**	UK (Wales)		Penwyllt Cave			Late Glacial	1a1	1a				1	
**RR3.3**	Spain		Sima de Illobi	8570 ± 40 (OxA-30296)	9538 ± 14		1a14	1a	-19.7	5.5	1185	1	
**8SS**	Spain		Sima de Illobi	8053 ± 35 (OxA-30243)	8921 ± 91		1a21	1a	-19.6		1185	1	
**5SS**	Spain		Sima de Illobi	8010 ± 45 (OxA-30297)	8885 ± 92		1a25	1a	-19.1	6.6	1185	1	
	Spain	EF488487	Cuevas del Somo	7500±55	8304±67		1a21	1a				1	Valdiosera et al. 2007

**15SS**	Spain		Vitoria	7480 ± 50 (X-2586-7)	8293 ± 64		1a21	1a	-19.4			1	
**RR1**	Spain		Sima de Illobi	7069 ± 38 (OxA-30298)	7906 ± 37		1a25	1a	-19.8	5.2	1185	1	
**12SS**	Spain		Sima de Illobi	7063 ± 33 (OxA-30244)	7904 ± 34		1a21	1a	-19.8		1185	1	
**Sw1**	Sweden		Motala, kanaljorden			ca 7600	1a4	1a	-20.5	4.7	<100	1	
**17SS**	Spain		Vitoria	6397 ± 32 (OxA-30245)	7345 ± 52		1a23	1a	-20.0			1	
	Spain	EU400179	Burgos	6325±50	7254±57		1a21	1a				1	Valdiosera et al. 2008
**CR1**	Spain		Atxagakoa	6188 ± 35 (OxA-30240)	7091 ± 59		1a24	1a	-18.9	7.3	N/A	1	
**NS3**	The Netherlands		North Sea	5781 ± 33 (OxA-30158)	6586 ± 48		1a5	1a	-21.3	5.7	<100	1	
**UR.ET. 02**	Spain		Cueva del Trocs	5377 ± 34 (OxA-30299)	6545 ± 55		1a27	1a	-19.5	2.6	N/A	1	
**Bu16**	Bulgaria		Unknown loc./ P4,4	5665 ± 50 (OxA-32244)	6456 ± 53		1a31	1a	-19.6	5.2	N/A	1	
	Spain	EF488490	Cuevas del Somo	5380±45	6167±91		1a21	1a				1	Valdiosera et al. 2007
**Ro5**	Romania		Mehedinti mts.	5018 ± 34 (OxA-30259)	5784 ± 77		1a1	1a	-18.6			1	
	Spain	EF488503	Cuevas del Somo	4624±45	5381±62		1a21	1a				1	Valdiosera et al. 2007
	Ireland	JF900166	Leitrim	4520±37	5180±93		1a17	1a	-21.6	8.8	N/A	1	Edwards et al. 2011
**Sw20**	Sweden		Alvastra pålbyggnad			ca 5100	Partial	1a				1	
**Sw19**	Sweden		Grödinge/Korsnäs	4145 ± 40	4699 ± 90		1a9	1a				1	
	Ireland	JF900174	Sligo	4136±37	4687±94		1a16	1a	-21.1	4.6	N/A	1	Edwards et al. 2011
	Ireland	JF900167	Leitrim	3517±31	3791±50		1a30	1a	N/A	N/A		1	Edwards et al. 2011
**Bu9**	Bulgaria		Baley, N-W Bulgaria	3267 ± 27 (OxA-30133)	3505 ± 41		1a10	1a	-19.8	6.5	100	1	
	Ireland	JF900175	Leitrim	2956±33	3126±60		1a29	1a	-21.7	4.6	N/A	1	Edwards et al. 2011
	Scotland	AY082845/81	Bear Cave	2673±54	2801±80		1a5	1a	N/A	N/A		1	Barnes et al. 2002
**Sw9**	Sweden		Sotenäs kanal	1959 ± 27 (OxA-32193)	1916 ± 27		1a8	1a	-20.6	3.7	<100	1	
	France	EF488491	Mt. Ventoux	1790±55	1718±79		1a26	1a	-19.3	3.7	1650	1	Valdiosera et al. 2007
**Bu8**	Bulgaria		Svinskata cave	1783 ± 27 (OxA-30403)	1709 ± 59		1a32	1a	-19.2	4.3	450	1	
	France	EF488495	Mt. Ventoux	1570±35	1467±44		1a26	1a				1	Valdiosera et al. 2007
	Spain	EF488497	Navacepada	350±40	405±63		1a22	1a				1	Valdiosera et al. 2007
**GX22**	Norway		Skien	192 ± 26 (OxA-32236)	156 ± 124		1a2	1a	-19.6	6.8	<100	1	
	Spain	EU400207	Asturias			1910 AD	1a12	1a				1	Valdiosera et al. 2008

Sweden	KM886408	Sonfjället	1928 AD	1a13	1a	1	Xenikoudakis et al. 2015
Norway	KM886413	Southern part	1905 AD	1a2	1a	1	Xenikoudakis et al. 2015
Norway	KM886414	Telemark fylke	1907 AD	1a15	1a	1	Xenikoudakis et al. 2015
Norway	KM886415	Numedal	1905 AD	1a6	1a	1	Xenikoudakis et al. 2015
Norway	KM886416	Rendalen	1859 AD	1a13	1a	1	Xenikoudakis et al. 2015
Sweden	KM886417	Dalarna	1864 AD	1a13	1a	1	Xenikoudakis et al. 2015
Sweden	KM886418	Värmland	1832 AD	1a2	1a	1	Xenikoudakis et al. 2015
Sweden	KM886421	Dalarna	1864 AD	1a3	1a	1	Xenikoudakis et al. 2015
Sweden	KM886422	Jämtland	1846 AD	1a13	1a	1	Xenikoudakis et al. 2015
Sweden	KM886424	Dalarna	1864 AD	1a13	1a	1	Xenikoudakis et al. 2015
Sweden	KM886425	Jämtland	1842 AD	1a13	1a	1	Xenikoudakis et al. 2015
Sweden	KM886427	Dalarna	1864 AD	1a13	1a	1	Xenikoudakis et al. 2015
Sweden	KM886428	Dalarna	1864 AD	1a3	1a	1	Xenikoudakis et al. 2015
Sweden	KM886429	Nordansjö	1830 AD	1a1	1a	1	Xenikoudakis et al. 2015
Sweden	KM886430	Värmland	1832 AD	1a1	1a	1	Xenikoudakis et al. 2015
Norway	KM886431	Ulvdalen	1899 AD	1a2	1a	1	Xenikoudakis et al. 2015
Norway	KM886432	Hjartdal	1905 AD	1a6	1a	1	Xenikoudakis et al. 2015
Sweden	KM886437	Värmland	1835 AD	1a2	1a	1	Xenikoudakis et al. 2015
Sweden	KM886438	Dalarna	1869 AD	1a8	1a	1	Xenikoudakis et al. 2015
Norway	KM886439	Hjartdal	1905 AD	1a6	1a	1	Xenikoudakis et al. 2015
Norway	KM886440	Etnedal	1899 AD	1a2	1a	1	Xenikoudakis et al. 2015

Sweden	KM886441	Norrland		1865 AD	1a11	1a	1	Xenikoudakis et al. 2015
Sweden	KM886442	Värmland		1844 AD	1a5	1a	1	Xenikoudakis et al. 2015
Sweden	KM886443	Bohuslän		1845 AD	1a1	1a	1	Xenikoudakis et al. 2015
Sweden	KM886444	Värmland		1847 AD	1a5	1a	1	Xenikoudakis et al. 2015
Sweden	KM886445	Dalarna		1848 AD	1a1	1a	1	Xenikoudakis et al. 2015
Sweden	KM886446	Värmland		1880 AD	1a2	1a	1	Xenikoudakis et al. 2015
Sweden	KM886448	Värmland		1927 AD	1a2	1a	1	Xenikoudakis et al. 2015
Sweden	KM886449	Värmland		1927 AD	1a8	1a	1	Xenikoudakis et al. 2015
Sweden	KM886450	Västergötland		1838 AD	1a1	1a	1	Xenikoudakis et al. 2015
Norway	KM886451	Bergen		1895 AD	1a2	1a	1	Xenikoudakis et al. 2015
Sweden	KM886452	Närke		1860 AD	1a5	1a	1	Xenikoudakis et al. 2015
Sweden	KM886453	Dalsland		1840 AD	1a1	1a	1	Xenikoudakis et al. 2015
Sweden	KM886454	Värmland		1845 AD	1a1	1a	1	Xenikoudakis et al. 2015
Sweden	KM886455	Värmland		1800 AD	1a2	1a	1	Xenikoudakis et al. 2015
Sweden	KM886456	Värmland		1869 AD	1a8	1a	1	Xenikoudakis et al. 2015
Norway	KM886457	Unknown		1862 AD	1a6	1a	1	Xenikoudakis et al. 2015
Spain	X75865	Cantabria	modern		1a12	1a	1	Taberlet & Bouvet 1994
Spain	X75866	Cantabria	modern		1a12	1a	1	Taberlet & Bouvet 1994
Sweden	X75868	Dalarna	modern		1a13	1a	7	Taberlet & Bouvet 1994
Norway	X75871		modern		1a1	1a	1	Taberlet & Bouvet 1994
France	X75878	Pyrenées	modern		1a26	1a	4	Taberlet & Bouvet 1994
France	EU497665	Pyrenées	modern		1a28	1a	1	Bon et al. 2008

	Spain	EU400184-207	Cantabria	modern			1a12	1a				21	Valdiosera et al. 2008
	Spain	EU400197	Cantabria	modern			1a12	1a				1	Valdiosera et al. 2008
	Spain	EU400202	Cantabria	modern			1a19	1a				1	Valdiosera et al. 2008
	Spain	EU400205	Cantabria	modern			1a18	1a				1	Valdiosera et al. 2008
	Bulgaria	KJ638594		modern			1a33	1a				8	Frosch et al. 2014
	Sweden			modern			1a13	1a				20	Xenikoudakis et al. 2015
**Pa13**	Austria		Herdengel-cave	53000 ± 1700		53000	1b6	1b			878	1	
**G32**	Georgia		Kudaro 1 Cave, layer 3, Caucasus	>48600 (OxA-32245)	(>52722)		1b5	1b	-19.6	3.2	1600	1	
**NS17**	The Netherlands		North Sea	>48500 (OxA-32253)	(>52598)		1b12	1b	-19.8	9.9	<100	1	
**R5**	The Netherlands		North Sea	47100 ± 2900 (OxA-32256)	51555 ± 4035		1b2	1b	-19.7	8.5	<100	1	
**NS2**	The Netherlands		North Sea	47100 ± 2800 (OxA-30157)	51489 ± 3925		1(b)10	1b	-19.4	10.7	<100	1	
**NS20**	The Netherlands		North Sea	>46800 (OxA-32254)	(>50333)		1b8	1b	-19.8	10.0	<100	1	
**NS23**	The Netherlands		North Sea	44500 ± 2100 (OxA-32255)	48237 ± 2544		1b7	1b	-19.5	9.3	<100	1	
**Pa2**	Slovenia		Matjazeve kamer	>43500 (Beta-422073)	(>47102)		1b3	1b	-19.0	3.0	540	1	
	Austria	AJ809334	Winden	40020±1000	43833±874		1b1	1b				1	Hofreiter et al. 2004
	Ireland	JF900164	Cork	37870±1270	42618±628		1b2	1b	-20.2	6.0	100	1	Edwards et al. 2011
	Ireland	JF900163	Cork	33310±770	39055±1384		1b9	1b	-19.7	5.9	100	1	Edwards et al. 2011
	Ireland	JF900171	Cork	32648±245	37970±613		Partial	1b	-20.0	5.5	N/A	1	Edwards et al. 2011
**M1**	Austria		Mariannenhöhle	29475 ±174	33840 ± 302		1b12	1b			636	1	
**Mj6**	Belgium		Goyet, 3e caverne	28800 ± 320 (OxA-32250)	33277 ± 461		1b12	1b	-19.2	8.7	130	1	
**Pa14**	Austria		Repolust-cave	27270±220	31930 ± 179		1b1	1b			525	1	
**J16**	UK (Wales)		Little Hoyle			LGM?(>20kBP)	1b11	1b				1	
**Hu6**	Hungary		Szelim-barlang, B réteg, Komárom megye	13255 ± 60 (OxA-30262)	16185 ± 409		1b25	1b	-19.5			1	
**Hu9**	Hungary		Szelim-barlang, B réteg, Komárom megye	13240 ± 28 (OxA-30268)	16170 ± 399		1b20	1b	-19.8	4.6	292	1	
**J2**	Belgium		Trou al'Wesse	12800 ± 60 (OxA-30251)	15285 ± 256		1b25	1b	-20.0			1	
**J1**	Belgium		Trou al'Wesse	12670 ± 60 (OxA-30404)	15047 ± 307		1b25	1b	-19.8	4.4	200	1	

**Mj4**	Belgium		Furfooz, Trou du Frontal			Post-LGM	1b19	1b				1	
**Mj2**	Belgium		Trou de Chaleux			Post-LGM	1b25	1b				1	
**Pa10**	Austria		Gudenus cave	12480 ± 55 (OxA-30264)	14784 ± 299		1b25	1b	-19.5	5.0	496	1	
**Pl19*4**	Poland		Komarowa Cave	12260 ± 60	14346 ± 276		1b25	1b				1	
**Pl7**	Poland		Wschodnia Cave	12170 ± 70	14206 ± 236		1b25	1b				1	
**Pl2**	Poland		Północna Duża Cave			14-29 kBP	1b4	1b				1	
**Pl4**	Poland		Niedźwiedzia Cave			<14000	1b25	1b				1	
**Sl3**	Slovakia		Nizke Tatry Mts.	11665 ± 50 (OxA-30250)	13551 ± 134		1b25	1b			900	1	
**Pl17**	Poland		Dziadowa Skała Cave			ca 12000	1b25	1b				1	
**Pa26**	Germany		Schlüssellochhöhle	10055 ± 33	11585 ± 149		1b25	1b	-18.7	5.2	1275	1	
**Pl3**	Poland		Południowa Cave	9860 ± 50	11492 ± 98		1b25	1b				1	
**Pa22**	Austria		Laufenbergloch	9810 ± 70	11241 ± 51		1b25	1b	-20.3	1.3	1445	1	
**Pa32**	Romania		Pestera cu Ceata	8790 ± 35	9813 ± 74		1b32	1b				1	
**Bu3**	Bulgaria		Pirin mountain	8367 ± 39 (OxA-30270)	9391 ± 58		1b17	1b	-18.7	6.7	N/A	1	
**Pa29**	Switzerland		Bärenfalle, Silberen	7845 ± 75	8707 ± 136		1b25	1b			2070	1	
**Pa20**	Switzerland		Geissbachhöhle	6810 ± 50	7650 ± 36		1b25	1b			1260	1	
**Hu10**	Hungary		Varbó, Lambrecht Kálmán-barlang	6064 ± 32 (OxA-30699)	6925 ± 40		1b32	1b	-18.8	8.0	410	1	
**Hu11**	Hungary		Varbó, Lambrecht Kálmán-barlang	6038 ± 33 (OxA-30237)	6886 ± 50		1b32	1b	-18.0	8.8	410	1	
**Pa30**	Switzerland		Neuschneehöhle	5610 ± 70	6403 ± 69		1b25	1b			1680	1	
	Denmark	JQ823238		5310±20	6098±62		1b25	1b				1	Bray et al. 2013
**Pa27*3**	Switzerland		Bärenhöhle am Stoss	5260 ± 65	6060 ± 96		1b25	1b	-19.8	2.2	1165	1	
	Germany	EF488499	Bad Frankenhausen	5210±35	5965±28		1b25	1b				1	Valdiosera et al. 2007
**Sl2**	Slovakia		Západné Tatry Mts.	5003 ± 30 (OxA-30249)	5766 ± 80		1b14	1b			1133	1	
**Pl16**	Poland		Deszczowa Cave	4000± 100	4500± 170		1b25	1b				1	
**Bu1**	Bulgaria		Dolnoslav			ca. 5000	1b18	1b				1	
**Pl1**	Poland		Ruska Skała Rock Shelter	3935 ± 35	4545 ± 99		1b32	1b				1	
**G20**	Ukraine		Luka Vrobletski			ca. 4000	1b25	1b				1	

**Hu18**	Hungary		Varbó, Lambrecht Kálmán-barlang, P/I. réteg	3528 ± 30 (OxA-30213)	3801 ± 54		1b15	1b	-19.4	4.4	410	1	
**Hu15**	Hungary		Varbó, Lambrecht Kálmán-barlang, P/II. réteg	3499 ± 28 (OxA-30238)	3778 ± 45		1b15	1b	-20.0	4.2	410	1	
	France	EF488505	Mt. Ventoux	3445±40	3730±72		1b25	1b				1	Valdiosera et al. 2007
**Bu12**	Bulgaria		Mazeta/Mazeto cave	3216 ± 31 (OxA-30419)	3434 ± 28		1b31	1b	-18.5	4.0	1350	1	
**Pa31**	Austria		Eichberghöhle	2695 ± 45	2810 ± 37		1b23	1b			850	1	
**Aix3**	France		Doubs	2497 ± 26 (OxA-30247)	2605 ± 83		1b25	1b	-20.6			1	
**Pl22**	Poland		Cave in Tatra Mts.	2330 ± 30	2190 ± 30		1b32	1b				1	
**Hu12**	Hungary		Varbó, Lambrecht Kálmán-barlang, P/II. réteg	2052 ± 25 (OxA-30263)	2022 ± 36		1b25	1b	-18.9	7.8	410	1	
**Mz4**	Germany		Mainz			ca 1950	1b16	1b				1	
**Pl23**	Poland		Nad Zagonem Cave (Zielona Cave), Tatra Mts.			ca 1800	1b32	1b				1	
	France	EF488493	Mt. Ventoux	1750±30	1662±40		1b28	1b	-19.6	4.1	1650	1	Valdiosera et al. 2007
**Pa25**	Austria		Bärenfalle	1730 +-30	1643 ± 46		1b25	1b				1	
**Aix2**	France		Doubs	1723 ± 23 (OxA-30246)	1640 ± 45		1b25	1b	-19.8			1	
	Germany	EF488501	Dienstedt	1665±35	1580±38		1b32	1b				1	Valdiosera et al. 2007
**Pa21**	Austria		Wildes Loch	1210 ± 30	1137 ± 45		1b21	1b	-20.1	2.6	1796	1	
**Ro4**	Romania		Mehedinti mts.	1160 ± 25 (OxA-30215)	1078 ± 55		1b32	1b	-19.9	3.7	N/A	1	
**Ro3**	Romania		Mehedinti mts.	1109 ± 26 (OxA-30214)	1017 ± 32		1b34	1b	-20.5	2.2	N/A	1	
**H1**	Romania		Apuseni mts.	988 ± 24 (OxA-30134)	891 ± 24		1b22	1b	-19.5	5.5	N/A	1	
	Germany	EF488500	Wysburg	750±150	728±135		1b25	1b				1	Valdiosera et al. 2007
**Sl1**	Slovakia		Belianske Tatry Mts	456 ± 25 (OxA-30248)	515 ± 8		1b25	1b	-20.8		1548	1	
**Ro2**	Romania		Fagaras mts.	372 ± 24 (OxA-30257)	416 ± 67		1b32	1b				1	
**Bu15**	Bulgaria		District of Trun			ca. 200	1b13	1b				1	
**Bu6**	Bulgaria		Temnata dupka	180 ± 22 (OxA-30562)	149 ± 125		1b35	1b	-17.1	2.9	420	1	
**Ro10**	Romania		Fagaras mts.	125 ± 25 (OxA-30258)	139 ± 99		1b34	1b	-18.5			1	
**Pl18**	Poland		Grota Kasprowa, Tatra Mts.			ca 50	1b32	1b				1	
**Ro6**	Romania		Mehedinti mts.	1900 AD (OxA-30260)			1b12	1b	-20.8			1	

Italy	X75862	Abruzzo	modern	1b25	1b	4	Taberlet & Bouvet 1994
Bulgaria	X75864		modern	1b32	1b	1	Taberlet & Bouvet 1994
Croatia	X75867		modern	1b26	1b	1	Taberlet & Bouvet 1994
Greece	X75870		modern	1b31	1b	1	Taberlet & Bouvet 1994
Slovenia	X75877		modern	1b25	1b	13	Taberlet & Bouvet 1994
Croatia	X75877		modern	1b25	1b	1	Taberlet & Bouvet 1994
Bosnia	X75877		modern	1b25	1b	2	Taberlet & Bouvet 1994
Italy	X75877	Trentino	modern	1b25	1b	1	Taberlet & Bouvet 1994
Romania			modern	1b34	1b	1	Zachos et al. 2008
Romania			modern	1b32	1b	4	Zachos et al. 2008
Romania			modern	1b34	1b	1	Zachos et al. 2008
Romania			modern	1b32	1b	1	Zachos et al. 2008
Romania			modern	1b32	1b	1	Zachos et al. 2008
Romania			modern	1b33	1b	1	Zachos et al. 2008
Romania			modern	1b32	1b	3	Zachos et al. 2008
Italy			modern	1b25	1b	17	Zachos et al. 2008
Croatia	HQ602651		modern	1b25	1b	45	Kocijan et al. 2011
Croatia	HQ602652		modern	1b29	1b	13	Kocijan et al. 2011
Croatia	HQ602653		modern	1b26	1b	1	Kocijan et al. 2011
Bulgaria	AP012591		modern	1b35	1b	1	Hirata et al. 2013
Bulgaria	KJ638591		modern	1b34	1b	5	Frosch et al. 2014
Bulgaria	KJ638592		modern	1b35	1b	53	Frosch et al. 2014
Bulgaria	KJ638593		modern	1b36	1b	10	Frosch et al. 2014
Bulgaria			modern	1b32	1b	9	Frosch et al. 2014
Turkey		Akseki	modern	1b30	1b	1	Cilingir et al. 2015
Turkey		Bursa	modern	1b29	1b	1	Cilingir et al. 2015
Romania	HE657199		modern	1b27	1b	1	Hailer et al. 2012

	Romania	HE657200		modern			1b34	1b				1	Hailer et al. 2012
	Romania	HE657213		modern			1b34	1b				1	Hailer et al. 2012
	Romania	HE657214		modern			1b32	1b				1	Hailer et al. 2012
**Aix1**	France		Doubs			Holocene?	1b25	1b				1	
**Sl4**	Slovakia		Jaskyňa Izabely Textorisovej Cave, Velká Fatra Mts.			?	1b37	1b			754	1	
**Bu2**	Bulgaria		Dolnoslav. SM<br>1854			?	1b16	1b				1	
**Pa24**	Austria		Bärenhöhle im Stockerstein			?	1b25	1b				1	
**Mj8**	Belgium		Goyet, 3e caverne	16840 ± 90 (OxA-32252)	20063 ± 280		1c1	1c	-19.3	6.4	130	1	
**Mj5**	Belgium		Goyet, 3e caverne	12960 ± 65 (OxA-32248)	15783 ± 405		1c1	1c	-19.8	5.3	130	1	
**Pa34**	Switzerland		Wildenmannlisloch	10325 ± 50	12221 ± 171		1c2	1c			1620	1	
**Aix4**	France		Isère	8740 ± 45	9733 ± 100		1c2	1c				1	
	France	EF488494	Mt. Ventoux	6525±50	7434±50		1c2	1c				1	Valdiosera et al. 2007
	France	EF488496	Mt. Ventoux	4645±40	5391±57		1c4	1c				1	Valdiosera et al. 2007
	France	EF488492	Mt. Ventoux	3845±40	4276±85		1c3	1c	-19.7	3.7	1650	1	Valdiosera et al. 2007
**It1**	Italy		Columbare, Negrar Verona			Holocene site	Partial	1c				1	
	Lebanon	FN292971				1870 AD	1d1	1d				1	Calvignac et al. 2009
	Turkey		Akseki	modern			1d2	1d				1	Cilingir et al. 2015
	Spain	EU400181	Valdegoba		80 k*		1e.1	1e				1	Valdiosera et al. 2008
	Spain	EU400182	Valdegoba		80 k*		1e.3	1e				1	Valdiosera et al. 2008
	Spain	EU400183	Valdegoba	75-90 ka	80 k*		1e.2	1e				1	Valdiosera et al. 2008
	Spain	EF488504	Atapuerca	17440±425	20881±318		1e.1	1e				1	Valdiosera et al. 2007
	Ireland	JF900162	Cork	26340±32	30937±254		2.7	2	-21.5	9.5	N/A	1	Edwards et al. 2011
	Ireland	JF900160	Kesh Corran	11920±85	13683±182		Partial	2	-17.8	6.7	N/A	1	Edwards et al. 2011
	Ireland	JF900168	Kesh Corran	11460±57	13219±105		2.4	2	-17.9	6.5	N/A	1	Edwards et al. 2011
	Ireland	JF900159	Limerick	10650±100	12569±174		2.6	2	-21.2	7.0	N/A	1	Edwards et al. 2011
	Ireland	JF900170	Clare	10495±51	12393±199		2.5	2	-19.9	6.1	N/A	1	Edwards et al. 2011
	Ireland	JF900172	Clare	9946±53	11391±125		2.2	2	-18.5	9.4	N/A	1	Edwards et al. 2011
	Ireland	JF900169	Limerick	8719±48	9702±96		2.3	2	-21.4	6.8	N/A	1	Edwards et al. 2011
**J20**	UK (Wales)		Pontnewydd Cave			Pre-LGM	3.2	3				1	

**J23**	UK (Wales)		Pontnewydd Cave			Pre-LGM	3.2	3				1	
**J26**	UK (Wales)		Pontnewydd Cave			Pre-LGM	3.15	3				1	
**J29**	UK (Wales)		Pontnewydd Cave			Pre-LGM	3.1	3				1	
**J32**	UK (Wales)		Pontnewydd Cave			Pre-LGM	3.1	3				1	
**J33**	UK (Wales)		Pontnewydd Cave			Pre-LGM	3.24	3				1	
	Spain	EU400180	Arlanpe	40-80 ka	60 k*		3.26	3				1	Valdiosera et al. 2008
	Austria	AJ809333	Ramesch	47420±1000	51184 ± 2166		3.23	3				1	Hofreiter et al. 2004
**Pl9**	Poland		Biśnik Cave	>50000	>50000		3.1	3				1	
**NS5**	The Netherlands		North Sea	>44500 (OxA-30159)	(>47895)		3.18	3	-19.5	6.2	<100	1	
	Ireland	JF900165	Waterford	32430±670	37818±915		2.1	2	-18.7	7.2	N/A	1	Edwards et al. 2011
**J27**	UK (Wales)		Pontnewydd Cave	32900 ± 800	37490 ± 1175		3.1	3				1	
	Germany	FR733658	Geissenklösterle	30560±180	35008±358		Partial	3	-19.5	8.3	580	1	Münzel et al. 2011
	Germany	FR733658	Geissenklösterle	30320±190	34876±276		Partial	3	-19.3	9.1	580	1	Münzel et al. 2011
	Ireland	JF900173	Waterford	28390±177	33067±625		2.1	2	-19.1	7.6	N/A	1	Edwards et al. 2011
**J30**	UK (Wales)		Pontnewydd Cave	28400 ± 300 (OxA-32247)	32829 ± 423		3.1	3	-19.6	10.6	<100	1	
**Pa11**	Austria		Krems-Wachtberg	26800 ± 220	31561 ± 251		3.1	3			260	1	
	Ireland	JF900161	Cork	26340±32	30937±254		Partial	2	-16.1	9.6	N/A	1	Edwards et al. 2011
**J17**	UK (Wales)		Little Hoyle	23010 ± 180 (OxA-32246)	27579 ± 450		3.1	3	-17.7	8.7	<100	1	
**Pa9**	Czech Rep.		Pod hradem	21940 ± 140 (OxA-30136)	26330 ± 382		3.1	3	-19.5	6.9	N/A	1	
	Germany	FR733658	Hohle Fels	14600±60	17743±286		Partial	3			450	1	Münzel et al. 2011
**Pl11**	Poland		Sokole Mts			<14000	3.1	3				1	
**J36**	UK (Wales)		Priory Farm Cave			Late Glacial	3.1	3				1	
**Sw12**	Sweden		Årnarp, Halland	8955 ± 45 (OxA-32193)	10081 ± 107		3.13	3	-20.7	4.8	<100	1	
**Sw11**	Sweden		Sturefors, Östergötland	8040 ± 30 (Beta-419016)	8913 ± 92		3.17	3	-19.9	5.1	<100	1	
	Norway	JQ823242		6210±100	7109±123		3.5	3				1	Bray et al. 2013
**G9**	Estonia		Unknown	5628 ± 33 (OxA-30300)	6400 ± 47		3.1	3	-21.2	5.1	N/A	1	
**Sw13**	Sweden		Erikslund, Sillre, Medelpad	5443 ± 30 (X-2581-54)	6254 ± 31		3.16	3	-20.2	3.4	<100	1	
	Norway	JQ823252		4420±70	5072±148		3.1	3				1	Bray et al. 2013
**Hu20**	Hungary		Peskő-barlang, humusz	4017 ± 31 (OxA-30239)	4484 ± 36		3.9	3	-18.8	8.4	745	1	

	Norway	JQ823256		3980±180	4451±263		3.1	3		1	Bray et al. 2013
	Norway	JQ823257		3970±60	4422±91		3.1	3		1	Bray et al. 2013
	Norway	JQ823245		3530±110	3827±141		3.1	3		1	Bray et al. 2013
	Norway	JQ823240		3150±120	3359±149		3.1	3		1	Bray et al. 2013
	Norway	JQ823244		2870±80	3022±117		3.1	3		1	Bray et al. 2013
	Norway	JQ823243		2800±160	2974±190		3.1	3		1	Bray et al. 2013
**G7**	Russia		European Russia			2800-3000	3.7	3		1	
**G6**	Russia		Urals			2300-2700	3.4	3		1	
	Romania	EF488506	Pestera Baltagul	2250±50	2253±69		3.9	3		1	Valdiosera et al. 2007
	Romania	EF488507	Pestera Baltagul	2250±50	2253±69		3.9	3		1	Valdiosera et al. 2007
**G15**	Russia		European Russia			ca 2200	3.25	3		1	
	Germany	EF488498	Mühlberg	1770±35	1690±54		3.1	3		1	Valdiosera et al. 2007
	Norway	JQ823250		1310±30	1243±40		3.7	3		1	Bray et al. 2013
**G3**	Russia		Kama River			ca. 1100	3.21	3		1	
**Sw8**	Finland		Enontek iö näkkälä	830 ± 25	740 ± 27		3.22	3		1	
**Ro8**	Romania		Southern Carpathians	188 ± 23 (OxA-30261)	153 ± 125		3.7	3	-20.0	1	
**GX47**	Norway		Karlebotn		(1880 AD)		3.22	3		1	
**Sw37**	Norway		Alten		(1894 AD)		3.22	3		1	
	Sweden	KM886400	Arjeplog		1939 AD		3.1	3		1	Xenikoudakis et al. 2015
	Sweden	KM886401	Jokkmokk		1939 AD		3.1	3		1	Xenikoudakis et al. 2015
	Sweden	KM886402	Porjus		1939 AD		3.1	3		1	Xenikoudakis et al. 2015
	Sweden	KM886403	Dorotea		1940 AD		3.2	3		1	Xenikoudakis et al. 2015
	Sweden	KM886404	Murjek		1940 AD		3.1	3		1	Xenikoudakis et al. 2015
	Sweden	KM886405	Kåitokvagga		1940 AD		3.2	3		1	Xenikoudakis et al. 2015
	Sweden	KM886406	Dorotea		1940 AD		3.2	3		1	Xenikoudakis et al. 2015
	Sweden	KM886407	Norråker		1940 AD		3.2	3		1	Xenikoudakis et al. 2015

Sweden	KM886409	Paittasjärvi		1928 AD	3.1	3	1	Xenikoudakis et al. 2015
Sweden	KM886410	Gällivare		1932 AD	3.1	3	1	Xenikoudakis et al. 2015
Sweden	KM886411	Kiruna		1910 AD	3.1	3	1	Xenikoudakis et al. 2015
Norway	KM886412	Hatfjelddalen		1915 AD	3.2	3	1	Xenikoudakis et al. 2015
Sweden	KM886419	Avaträsk		1915 AD	3.7	3	1	Xenikoudakis et al. 2015
Sweden	KM886420	Jämtland		1832 AD	3.1	3	1	Xenikoudakis et al. 2015
Sweden	KM886423	Kaalasluspa		1909 AD	3.1	3	1	Xenikoudakis et al. 2015
Sweden	KM886426	Vuoggatjålme		1909 AD	3.1	3	1	Xenikoudakis et al. 2015
Sweden	KM886433	Dalarna		1835 AD	1.1	3	1	Xenikoudakis et al. 2015
Sweden	KM886434	Vilhelmina		1939 AD	3.2	3	1	Xenikoudakis et al. 2015
Sweden	KM886435	Jämtland		1922 AD	3.2	3	1	Xenikoudakis et al. 2015
Sweden	KM886436	Gällivare		1940 AD	3.1	3	1	Xenikoudakis et al. 2015
Estonia	X75869		modern		3.12	3	2	Taberlet & Bouvet 1994
Romania	X75872		modern		3.19	3	1	Taberlet & Bouvet 1994
Romania	X75873		modern		3.9	3	3	Taberlet & Bouvet 1994
Russia	X75874		modern		3.1	3	1	Taberlet & Bouvet 1994
Estonia	X75874		modern		3.1	3	2	Taberlet & Bouvet 1994
Finland	X75874		modern		3.1	3	2	Taberlet & Bouvet 1994
Sweden	X75874	Norrland	modern		3.1	3	7	Taberlet & Bouvet 1994
Slovakia	X75875		modern		3.1	3	1	Taberlet & Bouvet 1994
Slovakia	X75876		modern		3.1	3	1	Taberlet & Bouvet 1994
Romania			modern		3.1	3	1	Zachos et al. 2008

Romania			modern	3.7	3	1	Zachos et al. 2008
Romania			modern	3.9	3	2	Zachos et al. 2008
Russia	AP012588	Urals	modern	3.1	3	1	Hirata et al. 2013
Bulgaria	AP012590		modern	3.9	3	1	Hirata et al. 2013
Bulgaria	KJ638595		modern	3.8	3	4	Frosch et al. 2014
Bulgaria	KJ638596		modern	3.10	3	1	Frosch et al. 2014
Bulgaria	KJ638597		modern	3.20	3	2	Frosch et al. 2014
Bulgaria			modern	3.9	3	8	Frosch et al. 2014
Bulgaria			modern	3.1	3	1	Frosch et al. 2014
Bulgaria			modern	3.7	3	1	Frosch et al. 2014
Estonia	EU526765		modern	3.1	3	1	Korsten et al. 2009
Finland	EU526765		modern	3.1	3	6	Korsten et al. 2009
Russia	EU526765		modern	3.1	3	52	Korsten et al. 2009
Russia	EU526766		modern	3.1	3	1	Korsten et al. 2009
Finland	EU526767		modern	3.14	3	2	Korsten et al. 2009
Russia	EU526768		modern	3.1	3	1	Korsten et al. 2009
Russia	EU526769		modern	3.7	3	1	Korsten et al. 2009
Finland	EU526770		modern	3.1	3	4	Korsten et al. 2009
Russia	EU526770		modern	3.1	3	4	Korsten et al. 2009
Russia	EU526771		modern	3.1	3	2	Korsten et al. 2009
Russia	EU526772		modern	3.1	3	7	Korsten et al. 2009
Finland	EU526773		modern	3.1	3	1	Korsten et al. 2009
Russia	EU526774		modern	3.1	3	1	Korsten et al. 2009
Russia	EU526776		modern	3.1	3	2	Korsten et al. 2009
Finland	EU526777		modern	3.1	3	7	Korsten et al. 2009
Russia	EU526777		modern	3.1	3	1	Korsten et al. 2009
Finland	EU526778		modern	3.3	3	1	Korsten et al. 2009
Finland	EU526779		modern	3.1	3	1	Korsten et al. 2009
Finland	EU526780		modern	3.1	3	1	Korsten et al. 2009
Russia	EU526781		modern	3.1	3	3	Korsten et al. 2009

	Russia	EU526782		modern		3.6	3	1	Korsten et al. 2009
	Russia	EU526783		modern		3.1	3	3	Korsten et al. 2009
	Estonia	EU526784		modern		3.1	3	27	Korsten et al. 2009
	Estonia	EU526785		modern		3.1	3	4	Korsten et al. 2009
	Russia	EU526785		modern		3.1	3	2	Korsten et al. 2009
	Russia	EU526786		modern		3.1	3	1	Korsten et al. 2009
	Russia	EU526787		modern		3.1	3	1	Korsten et al. 2009
	Russia	EU526788		modern		3.1	3	3	Korsten et al. 2009
	Russia	EU526789		modern		3.1	3	1	Korsten et al. 2009
	Russia	EU526791		modern		3.1	3	3	Korsten et al. 2009
	Finland	EU526792		modern		3.1	3	3	Korsten et al. 2009
	Finland	EU526793		modern		3.1	3	1	Korsten et al. 2009
	Russia	EU526793		modern		3.1	3	2	Korsten et al. 2009
	Finland	EU526799		modern		3.11	3	1	Korsten et al. 2009
	Estonia	EU526801		modern		3.1	3	2	Korsten et al. 2009
	Estonia	EU526802		modern		3.12	3	1	Korsten et al. 2009
	Bulgaria	HE657201		modern		3.1	3	1	Hailer et al. 2012
	Romania	HE657203		modern		3.1	3	1	Hailer et al. 2012
	Norway	HE657208		modern		3.22	3	1	Hailer et al. 2012
	Norway	HE657209		modern		3.22	3	1	Hailer et al. 2012
	Romania	HE657210		modern		3.7	3	1	Hailer et al. 2012
	Romania	HE657211		modern		3.9	3	1	Hailer et al. 2012
	Sweden	HE657212		modern		3.2	3	1	Hailer et al. 2012
	Norway	HE657215		modern		3.22	3	1	Hailer et al. 2012
	Norway	HE657216		modern		3.22	3	1	Hailer et al. 2012
	Sweden			modern		3.1	3	13	Xenikoudakis et al. 2015
	Sweden			modern		3.2	3	14	Xenikoudakis et al. 2015
**NS19**	The Netherlands		North Sea		LP-Early Holocene	Partial	3	1	
**G13**	Russia		Urals		Late Pleistocene	Partial	3	1	

	Norway			Holocene	3.1	3		10	Bray et al. 2013
	Sweden	KM886458	Nedre Vaptsjön	Medieval	3.1	3		1	Xenikoudakis et al. 2015
	Sweden	KM886459	Unna Saiva	Medieval	3.1	3		1	Xenikoudakis et al. 2015
